# Fine-mapping of a major locus for Fusarium wilt resistance in flax (*Linum usitatissimum* L.)

**DOI:** 10.1007/s00122-023-04528-2

**Published:** 2024-01-21

**Authors:** S. Cloutier, T. Edwards, C. Zheng, H. M. Booker, T. Islam, K. Nabetani, H. R. Kutcher, O. Molina, F. M. You

**Affiliations:** 1 Ottawa Research and Development Centre, Agriculture and Agri-Food Canada, 960 Carling Avenue, Ottawa, ON K1A 0C6 Canada; 2 Crop Development Centre, University of Saskatchewan, 51 Campus Drive, Saskatoon, SK S7N 5A8 Canada; 3 Morden Research and Development Centre, Agriculture and Agri-Food Canada, 101 Route 100, Morden, MB R6M 1Y5 Canada; 4 Present Address: Department of Plant Agriculture, Ontario Agricultural College, University of Guelph, 50 Stone Road E, Guelph, ON N1G 2W1 Canada

## Abstract

**Key message:**

Fine-mapping of a locus on chromosome 1 of flax identified an S-lectin receptor-like kinase (SRLK) as the most likely candidate for a major Fusarium wilt resistance gene.

**Abstract:**

Fusarium wilt, caused by the soil-borne fungal pathogen *Fusarium oxysporum* f. sp. *lini*, is a devastating disease in flax. Genetic resistance can counteract this disease and limit its spread. To map major genes for Fusarium wilt resistance, a recombinant inbred line population of more than 700 individuals derived from a cross between resistant cultivar ‘Bison’ and susceptible cultivar ‘Novelty’ was phenotyped in Fusarium wilt nurseries at two sites for two and three years, respectively. The population was genotyped with 4487 single nucleotide polymorphism (SNP) markers. Twenty-four QTLs were identified with IciMapping, 18 quantitative trait nucleotides with 3VmrMLM and 108 linkage disequilibrium blocks with RTM-GWAS. All models identified a major QTL on chromosome 1 that explained 20–48% of the genetic variance for Fusarium wilt resistance. The locus was estimated to span ~ 867 Kb but included a ~ 400 Kb unresolved region. Whole-genome sequencing of ‘CDC Bethune’, ‘Bison’ and ‘Novelty’ produced ~ 450 Kb continuous sequences of the locus. Annotation revealed 110 genes, of which six were considered candidate genes. Fine-mapping with 12 SNPs and 15 Kompetitive allele-specific PCR (KASP) markers narrowed down the interval to ~ 69 Kb, which comprised the candidate genes *Lus10025882* and *Lus10025891*. The latter, a G-type S-lectin receptor-like kinase (SRLK) is the most likely resistance gene because it is the only polymorphic one. In addition, Fusarium wilt resistance genes previously isolated in tomato and *Arabidopsis* belonged to the SRLK class. The robust KASP markers can be used in marker-assisted breeding to select for this major Fusarium wilt resistance locus.

**Supplementary Information:**

The online version contains supplementary material available at 10.1007/s00122-023-04528-2.

## Introduction

Flax (*Linum usitatissimum* L.) is one of the few species with multiple distinct plant parts with economic value, namely its stem and seed (Cloutier 2016). High-quality bast fiber used in the weaving of linen and in the production of fine paper and composites are extracted from the stem of fiber flax. In contrast, the flaxseed (or linseed) morphotype is grown for seed rather than fiber and this seed’s uniqueness resides in its composition, i.e., 40–50% oil that is rich in the omega-3 alpha-linolenic acid (55–60%) (Westcott and Muir 2003). This attribute forms the basis of the blood cholesterol lowering health claim granted to flaxseed by regulatory agencies (https://www.canada.ca/content/dam/hc-sc/migration/hc-sc/fn-an/alt_formats/pdf/label-etiquet/claims-reclam/assess-evalu/flaxseed-graines-de-lin-eng.pdf), and is responsible for the oil’s properties that makes it suitable for many industrial applications such as paints and varnishes. It is the multiple uses and versatility of flax that earned it the moniker *usitatissimum*, which means most useful.

Flaxseed (linseed), fiber flax and dual-purpose flax cultivars all belong to the same species and, as such, are affected by the same pathogens (Cullis 2019). A few major diseases plague flax production, and all are caused by fungi, namely flax rust (*Melamspora lini*), powdery mildew (*Oidium lini*), pasmo (*Septoria linicola*) and Fusarium wilt (*Fusarium oxysporum* f. sp. *lini*). The latter is the only one caused by a soil-borne pathogen. Once established in a soil, Fusarium wilt can survive for 20 years or more (Booker 2019). Crop rotation is an important control method to reduce the soil inoculum over time, but such agronomic practices have their limitations. Fungicide seed treatments can be effective but are costly, difficult to apply to flax seed and contaminate the environment with pesticides. The most effective and environmentally friendly method is to grow resistant cultivars in combination with crop rotation (Chitwood-Brown et al. 2021).

*Fusarium oxysporum* f. sp. *lini* (*Fol*) point of entry is the root system, from which it spreads via the xylem vascular system, hindering the normal water flow (Gordon 2017). Symptoms include yellowing and wilting of leaves in parts of the plant, to complete plant wilting and resulting in the death of the whole plant (Nehra et al. 2021). The early symptoms affect the topmost part of the plants, causing the wilted tip to fold down into an inverted “J” that resembles a shepherd’s crook. Because of the severity of the symptoms, Fusarium wilt can easily cause yield losses of 20%, and in the case of severe infection, entire fields can be lost (Rashid 2003).

Fusarium wilt has a wide host range and more than 120 *formae speciales* have been identified (Michielse and Rep 2009). Some of its most important host crops are tomato (*Solanum lycopersicum*), strawberry (*Fragaria* × *ananassa*), cotton (*Gossipium* spp.), banana (*Musa* spp.) and melon (*Cucumis melo*). Resistance to *F. oxysporum* infection seems to always be quantitative; however, major resistance genes have been isolated in tomato (Catanzariti et al. 2015, 2017; Gonzalez-Cendales et al. 2016; Ori et al. 1997; Simons et al. 1998), *Arabidopsis thaliana* (Cole and Diener 2013; Diener and Ausubel 2005), melon (Joobeur et al. 2004) and banana (Dale et al. 2017; Peraza-Echeverria et al. 2009). In flax, a few transcriptomics experiments have been conducted to identify the potential gene networks involved in the plant’s ability to mount a resistant response but no single major resistance gene has been identified (Boba et al. 2021; Dmitriev et al. 2017; Galindo-Gonzalez and Deyholos 2016).

The screening of a recombinant inbred line (RIL) population from a cross between the moderately resistant cultivar ‘Aurore’ and the susceptible cultivar ‘Oliver’ with two *Fol* isolates (Edirisinghe et al. 2023) indicated that resistance was controlled in this cross by two independently inherited recessive genes. Using a doubled haploid population derived from the susceptible cultivar ‘Glenelg’ and the resistant line CRZY8/RA91, Spielmeyer et al. (1998) mapped two quantitative trait loci (QTLs) for Fusarium wilt resistance in flax. Unfortunately, the mapping was performed using mostly amplified fragment length polymorphism (AFLP) markers and the location of the QTLs on linkage groups 6 and 10 cannot be assigned to the pseudomolecules of the flax reference genome assembly (You et al. 2018). Recently, Kanapin et al. (2021) performed a genome-wide association study (GWAS) with nearly 300 accessions and identified one major locus for Fusarium wilt resistance in flax on chromosome 1 and minor loci on chromosomes 8, 11 and 13. The major chromosome 1 locus comprised ten quantitative trait nucleotides (QTNs), spanned 640 Kb and explained 7–10% of the phenotypic variance for Fusarium wilt resistance in their diversity panel. Here, we used a 703-line biparental population derived from a cross between the resistant cultivar ‘Bison’ and the susceptible cultivar ‘Novelty’ to map Fusarium wilt resistance loci, and specifically to fine-map a major resistant locus on chromosome 1, toward the isolation of the causal gene.

## Materials and methods

### Plant material

‘Bison’ and ‘Novelty’ are flax cultivars that are resistant and susceptible checks for Fusarium wilt evaluation, respectively. ‘Bison’ and ‘Novelty’ were registered in Canada in 1930 and 1910, respectively. ‘Bison’ was bred in USA and has been used as a direct or an ancestral parent for 54 released cultivars (You et al. 2016). No pedigree record is available for either of these two cultivars. A cross between the two cultivars was made and a 703-RIL population (BN) was developed by single seed descent to the *F*_6_ generation. Half the RILs came from 15 ‘Bison’/’Novelty’ *F*_1_ plants and the other half were from 15 reciprocal ‘Novelty’/’Bison’ *F*_1_ plants.

### DNA and library preparation

All RILs, two *F*_1_s and both parental lines were grown in root trainers (72 plants per root trainer) in a growth chamber under 20 h light at 24 °C and 4 h dark at 18 °C. When seedlings were 3–5 cm tall, the compact leaf clump at the tip, ~ 50–80 mg of tissue, was sampled in Qiagen 96-well format collection tubes kept on liquid nitrogen. The tissue collected was then lyophilized in a freeze-dryer (FreeZone 6 L benchtop, Labconco, Kansas City, MO, USA) for 48–72 h until completely dry prior to storage at  − 20 °C until DNA extraction. Genomic DNA was extracted using the Qiagen DNeasy® kit (Qiagen, Toronto, ON, Canada) and quantified using the Quant-iT™ PicoGreen™ dsDNA assay kit (ThermoFisher Scientific, Waltham, MA, USA), both following manufacturer’s instructions. All DNA samples were diluted to 10 ng/µl.

Two hundred ng of genomic DNA was sent to the Institut de biologie intégrative et des systèmes (IBIS) at the Université Laval (Quebec, QC, Canada) for library construction. Eight 96-well plates were used, where the first two had 96 samples and the other six contained 88 samples for a total of 720 libraries. Each plate had an empty (water only) control well randomly positioned within each plate and either a parent or an *F*_1_. In all, three independent libraries of each parent and one of each of the reciprocal *F*_1_ plants were constructed. The remaining 704 libraries were constructed from the DNA of the RILs.

Library construction was performed following the protocol described in Colston-Nepali et al. (2019) but *Pst*I was used instead of *Sbf*I. Briefly, the DNA cut with restriction enzymes *Pst*I and *Msp*I was ligated to modified *Msp*I adapters to allow plate barcoding. Size-selected samples were amplified and barcoded using TruSeq HT primers (Illumina, San Diego, CA, USA). The 720 uniquely barcoded libraries were pooled and sequenced on one flow cell on the NovaSeq 6000 S4 platform using the 150-bp paired-end (PE) mode by the Centre d’expertise et de services Génome Québec (Montreal, QC, Canada).

### Data processing

The Illumina reads from each individual in the population were aligned to the flax reference genome sequence v2.0 (You et al. 2018) using BWA v0.6.1 (Jo and Koh 2015). The alignment process utilized a base-quality Q score in Phred scale > 20 and default parameters. The resulting alignment file for each individual was then employed as input for single nucleotide polymorphism (SNP) discovery, which was performed with the software package SAMtools v1.12 (Li et al. 2009).

To enhance the reliability of the SNP dataset, filtering was performed using the AGSNP pipeline (You et al. 2011, 2012) and its updated version (Kumar et al. 2012). Only SNPs with a minor allele frequency (MAF) > 0.05 and a minimum call rate > 80% were retained.

### Phenotyping for Fusarium wilt resistance

The BN RIL population was grown in established Fusarium wilt nurseries at five site-years: Morden (MB, Canada) in 2019 and 2021 and near Saskatoon (SK, Canada) from 2019 to 2021. Selected flax lines were seeded each year between June 3rd and June 7th in the Saskatoon and Morden flax wilt nurseries. The field conditions were warm and dry in both Saskatoon and Morden in all years, meaning above average temperatures and below average precipitation for the growing seasons at both sites, respectively. The experiments were organized in a type 2 modified augmented design (MAD 2) with one replicate. This design uses uniformly distributed control plots in the center of whole plots and randomly distributed control subplots to assess heterogeneity across the nursery without replication of individual lines. The single-row plot Fusarium wilt nurseries were planted using 2 g of seed (approximately 350 seeds) per 1.2 m row in Saskatoon and 3 g of seed (approximately 525 seeds) per 2 m row in Morden with row spacing of 30 cm. Fusarium wilt phenotyping is based on a combination of wilt symptoms and plant vigor assessments which include discoloration of leaves, reduction in height, reduction in branching, percentage of severely infected and dead plants. Assessment of disease severity, stand and vigor were done at three growth stages: seedling, stem elongation/early flowering and flowering/green bolls. Stand and vigor assessments were done based on a 1–5 scale where 1 = full stand and most vigorous and 5 = poor stand (< 10% of row) and least vigorous (Rashid and Kenaschuk 1993). Disease severity was assessed based on a 0–9 scale: 0 = no sign of wilt, vigorous flax growth; 9 = all plants severely wilted or dead as previously described (Rashid and Kenaschuk 1993). For each stage, Fusarium wilt severity corresponded to a single rating for all plants within a row and final rating was obtained by averaging over all three stage ratings.

### Quantitative trait locus (QTL) analyses

The QTL analysis for Fusarium wilt resistance was performed on seven phenotypic datasets: five individual site-years, the five site-year means, and the best linear unbiased predictors (BLUPs) estimated for the five site-years. Three different statistical models were employed for QTL mapping: linkage map-based, haplotype block-based and SNP-based multi-locus as described below.

To identify QTLs using segregation population information, a genetic map was constructed using QTL IciMapping v4.2 (Meng et al. 2015) with 4487 SNP markers, which were obtained using the filtering criteria of a MAF > 0.05 and a minimum call rate > 80%. After conducting a segregation distortion test and removing redundant markers, 2379 SNP markers were de novo mapped into 15 linkage groups based on recombination frequency. Genetic map distances were estimated using the Kosambi mapping function (Kosambi 1944). The resulting genetic map was used to detect QTLs with additive effect using the inclusive composite interval mapping (ICIM-ADD) model. Permutation tests with 1000 iterations and a type I error *α* = 0.05 were performed to determine the LOD scores to be used as thresholds of significance for QTL detection for each phenotypic dataset.

The RTM-GWAS (restricted two-stage, multi-locus, multi-allele GWAS) v2020.0 (He et al. 2017) haplotype block-based method was also employed to identify QTLs. The RTM-GWAS method initially groups SNPs exhibiting strong linkage disequilibrium (LD) (*D*′ > 0.8) into LD blocks that are subsequently utilized for QTL detection. A significance level of 0.05 was applied for the pre-selection of individual candidate markers using the single-locus model, while an experiment-wide significance level of 0.05 was used for stepwise regression to identify significant QTLs under the multi-locus model.

Finally, the multi-locus 3VmrMLM model was utilized to detect main effect QTNs. The R package IIIVmrMLM was employed with the following parameters: method = “Single_env”, SearchRadius = 20 and svpal = 0.01. The association signals of the 3VmrMLM model were identified using a LOD score ≥ 3 (Li et al. 2022). Additional statistical analyses were applied to the putative QTNs to remove those with no allelic effect. The Kruskal–Wallis nonparametric test was conducted with the R function “kruskal.test” for all putative QTN allele pairs using a significance threshold of 5% probability for retention. *R*^2^ values were calculated based on simple regressions of QTNs on Fusarium wilt ratings, representing the proportion of the total variation in Fusarium wilt resistance explained by the QTNs/QTLs.

### Candidate gene analyses

The genome-wide resistance gene analogs (RGAs) of the flax reference genome assembly ‘CDC Bethune’ v2.0 (You et al. 2018) were predicted using the RGAugury pipeline (Li et al. 2016), resulting in the identification of 1327 RGAs (You et al. 2018). Subsequently, all RGAs within the major QTL target region of chromosome 1 underwent further curation. Furthermore, the protein sequences of all annotated genes within the target region were subjected to a BLAST search against the NCBI protein database to identify potential disease-related genes that were not initially included in the RGAs predicted by RGAugury.

### Genome sequencing

The ~ 866 Kb region on chromosome 1 of the ‘CDC Bethune’ reference genome assembly that harbors a major QTL for Fusarium wilt resistance contained a large unresolved region of ~ 400 Kb, one of ~ 25 Kb and a few other small gaps. To resolve these regions, we sampled 3 g of tissue on liquid nitrogen and performed high molecular weight (HMW) genomic DNA extraction from deep-frozen tissue of ‘CDC Bethune’, ‘Bison’ and ‘Novelty’ using NucleoBond® HMW DNA kit (DMark Biosciences, Toronto, ON, Canada) as per manufacturer’s instructions. The DNA was quantified using Quant-iT™ PicoGreen™ dsDNA assay kit as described above. Quality was assessed by pulse-field gel electrophoresis using aliquots of HMW genomic DNA resolved against the HMW DNA standards Lambda PFG ladder and MidRange PFG marker (New England BioLabs, Ipswich, MA, USA). DNA samples ranging from 50 to more than 200–300 Kb were considered high quality. Approximately, 10–15 µg of high-quality HMW DNA (150–200 ng/µl) of each sample was sent to the Centre d’expertise et de services Génome Québec for library construction and PacBio long-read HiFi sequencing.

For each of the three genotypes, one PacBio sheared gDNA HiFi library was constructed, and each library was sequenced on a single PacBio Sequel II SMRT Cell (30 h). DNA libraries were prepared following the Pacific Biosciences protocol detailed in “Preparing whole genome and metagenome libraries using SMRTbell® prep kit 3.0” (Pacific Biosciences, Menlo Park, CA, USA). Briefly, HMW purified genomic DNA was sheared using the Diagenode Megaruptor 3 instrument (Diagenode Inc., Denville, NJ, USA). The DNA damage repair, end repair and SMRT bell ligation steps were performed as described in the template preparation protocol with the SMRTbell template prep Kit 3.0 reagents (Pacific Biosciences). The DNA libraries were size selected using AMPure PB bead procedure (Pacific Biosciences). The sequencing primer was annealed with sequencing primer 3.2 and the Sequel II 2.2 polymerase was bound to the template. The libraries went through a SMRTbell Cleanup bead (Pacific Biosciences) prior to sequencing on a PacBio Sequel II instrument at a loading concentration of 80 pM using the adaptive loading protocol, Sequel II Sequencing Kit 2.0, SMRT Cell 8 M and 30 h movies with a pre-extension time of 2 h.

### Genome assemblies

The HiFi consensus circular sequence (ccs) reads of ‘CDC Bethune’, ‘Bison’ and ‘Novelty’ were assembled using Hifiasm v0.13-r308 (Cheng et al. 2021) with default parameters. Redundant haplotigs were subsequently removed based on the coverage depth of HiFi ccs reads on assembled contigs using purge_dups v1.2.6 (Guan et al. 2020). The purged contigs were then mapped to the flax chloroplast genome sequence (NC036356) using minimap2 2.24-r1122 (Li 2018) to remove the contigs belonging to the chloroplast genome.

To merge contigs into super contigs, the purged and cleaned contigs were scaffolded using LRScaf v1.1.12 (Qin et al. 2019) utilizing the HiFi ccs reads as a reference. In the case of ‘CDC Bethune’, the de novo optical BioNano genome (BNG) maps of ‘CDC Bethune’ were aligned with the in silico BNG maps of scaffolds generated using the script fa2cmap_multi_color.pl in the BioNano Solve software (Bionano Genomics, San Diego, USA). The BNG contigs were employed to guide the order and orientation of the scaffold sequences, resulting in the generation of super scaffolds.

To generate chromosome-scale pseudomolecules, the super scaffolds were aligned to the already ordered and scaffolded chromosome-scale ‘CDC Bethune’ v2.0 assembly (You et al. 2018). This process yielded 15 chromosome-level sequences. Finally, the resulting scaffolds were polished using Inspector v1.0.2 (Chen et al. 2021) with the raw HiFi ccs reads.

### Marker development and fine-mapping

Alignment of the ‘Bison’ and ‘Novelty’ chromosome 1 target region revealed numerous SNPs and indels. Several SNPs spanning the target locus were selected and Kompetitive allele-specific PCR (KASP) assays were designed by LGC (Teddington, Middlesex, UK). The markers were resolved on the DNA of RILs with putative recombination within the target locus using LGC’s recommended conditions for each assay.

## Results

### SNP dataset and genetic map construction

After deplexing and filtering, an average of ~ 4.16 M 150 bp paired-end (PE) reads per individual was generated. Using a genome size estimate for flax of ~ 480 Mb, this read depth corresponds to an average of 1.3X coverage per individual. The initial read mapping performed against the ‘CDC Bethune’ reference genome assembly (You et al. 2018) yielded 4487 SNPs after filtering with MAF > 0.05 and call rate > 80%. A genetic map was constructed using the 2379 SNPs after removal of distorted and redundant markers. The de novo genetic map included 2374 SNPs that spanned 4153 cM with 96–282 SNPs per chromosome (Supplementary Fig. S1 and Table S1).

### Phenotyping

**Fig. 1:**
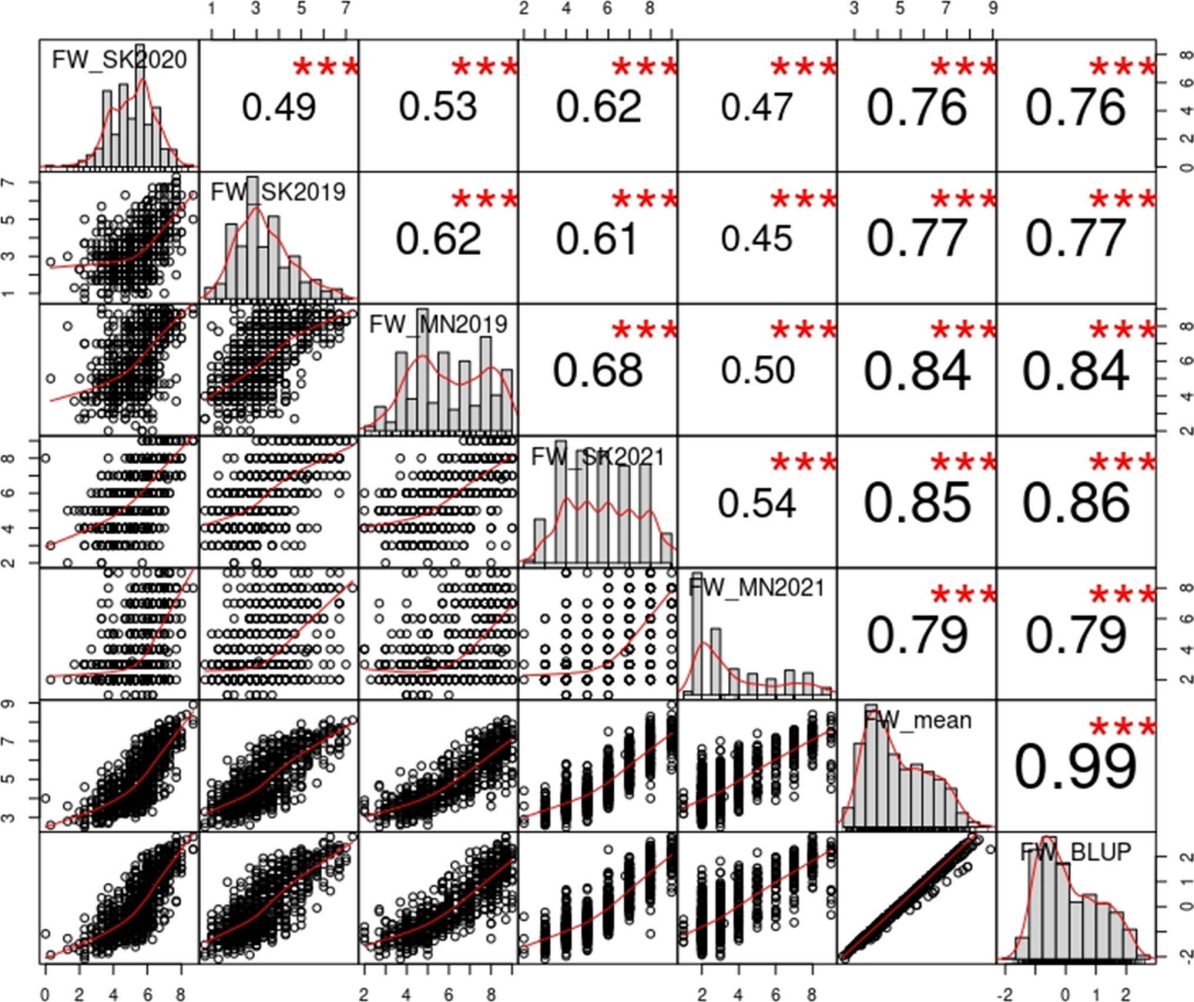
Distribution and correlation of the Fusarium wilt severity scores in flax (*Linum usitatissimum*) for the five site-years, means and best linear unbiased predictor (BLUP) values. Distributions of each dataset are on the diagonal. Correlations among datasets are illustrated below the diagonal and correlation values are above it. The site-years are Saskatoon 2019, 2020 and 2021, and Morden 2019 and 2021. FW_mean and FW_BLUP are the means and BLUP values of the Fusarium wilt severity scores across all five site-years in a recombinant inbred line (RIL) population derived from a cross between ‘Bison’ and ‘Novelty’. *** *P* value < 0.001

Fusarium wilt severity was scored on a 0–9 scale (0 = healthy and vigorous to 9 = severely wilted or dead) from all field trials grown in established Fusarium wilt nurseries near Saskatoon in 2019, 2020 and 2021 and in Morden in 2019 and 2021. The distribution of severity ratings was wide in all years, and Pearson’s correlation coefficients (*r*) between site-years were all significant, ranging from 0.45 to 0.68, while correlations between site-years and mean or BLUP ranged from 0.76 to 0.86 (Fig. 1). Correlations between mean and BLUP was 0.99, indicating that QTL mapping performed with either value would likely identify the same loci (Fig. 1). BLUP distribution by range clearly showed transgressive segregants at both ends (Supplementary Fig. S2).

### Quantitative trait locus (QTL) mapping and candidate genes

The QTL mapping of the Fusarium wilt severity score from all five site-years, the means over site-years and the BLUP values, performed using the recombination-based model ICIM of IciMapping, identified 24 QTLs. The GWAS model 3VmrMLM identified 18 quantitative trait nucleotides (QTNs) and the haplotype block-based model RTM-GWAS identified 108 LD blocks (Supplementary Table S2). QTLs for Fusarium wilt means detected by two or all three of the models are summarized (Table 1). A major QTL explaining 20–48% of the variance was detected by all three models on chromosome 1 in a 731,327 bp interval, and was co-located to a second QTL, extending the region to 866,993 bp based on the ‘CDC Bethune’ reference genome assembly v2.0 (You et al. 2018). Considering the importance of this locus, fine-mapping and candidate gene searches were performed. Annotation of this region based on the ‘CDC Bethune’ reference genome assembly v2.0 identified 110 genes (*Lus10025832*-*Lus10025941*), of which six were hypothesized to be candidate genes for Fusarium wilt resistance based on their predicted function (Supplementary Table S3).

**Table 1:** Quantitative trait locus (QTL) intervals, quantitative trait nucleotides (QTNs) and/or linkage disequilibrium (LD) blocks identified by at least two of the three models used to detect associations with Fusarium wilt severity score means in the recombinant inbred line (RIL) population derived from a *Linum usitatissimum* cross between ‘Bison’ and ‘Novelty’

Chromosome	Start position	End position	Model^a^	QTL Interval/QTN/LD block	Significant trait (dataset)	*R*^*2*^ (%)
1	1,966,369	2,500,703	ICIM-ADD	Lu1_1966369_2500703	BLUP, MN2019, MN2021, SK2019, SK2020, SK2021	47.99
			3VmrMLM	Lu1_1966369	BLUP, MN2019, MN2021, SK2019, SK2020, SK2021	20.11
			RTM-GWAS	Lu1_LDB_1769377_1966369	BLUP, MN2019, MN2021, SK2019, SK2020, SK2021	40.66
1	2,500,703	2,636,369	3VmrMLM	Lu1_2500703	BLUP, SK2021	4.74
			RTM-GWAS	Lu1_LDB_2500703_2636369	BLUP, MN2019, MN2021, SK2019, SK2021	1.86
3	22,290,905	22,290,944	ICIM-ADD	Lu3_19965022_22290944	BLUP, MN2019, SK2019	0.91
			RTM-GWAS	Lu3_LDB_22290905_22290944	BLUP, SK2019, MN2019	1.68
4	13,800,341	13,723,558	ICIM-ADD	Lu4_13290133_14284199	BLUP, MN2019, SK2019	6.56
			3VmrMLM	Lu4_13800341	BLUP	1.34
9	18,020,087	18,325,581	ICIM-ADD	Lu9_18020087_18325581	BLUP, MN2019	1.78
			3VmrMLM	Lu9_18325581	BLUP, MN2019	2.05
12	18,416,660	18,448,659	ICIM-ADD	Lu12_18416660_18448659	BLUP	1.04
			3VmrMLM	Lu12_18448659	BLUP, MN2019, MN2021, SK2020	2.14
			RTM-GWAS	Lu12_18448659	BLUP, MN2019, MN2021, SK2020	3.91
			RTM-GWAS	Lu12_LDB_18855826_18870803	BLUP, MN2021, SK2020, SK2021	0.99
14	3,181,022	3,311,895	ICIM-ADD	Lu14_3181022_3311895	BLUP, MN2019, SK2019, SK2020, SK2021	2.60
			3VmrMLM	Lu14_3311895	BLUP, SK2019, SK2020, SK2021	2.11

Chromosome, start and end positions, model, trait and percent of the variance explained (*R*^*2*^) are listed

^a^ICIM-ADD = inclusive composite interval mapping—additive effect model implemented in QTL IciMapping; 3VmrMLM model implemented in the *R* package IIIVmrMLM

### Whole-genome sequencing

**Fig. 2:**
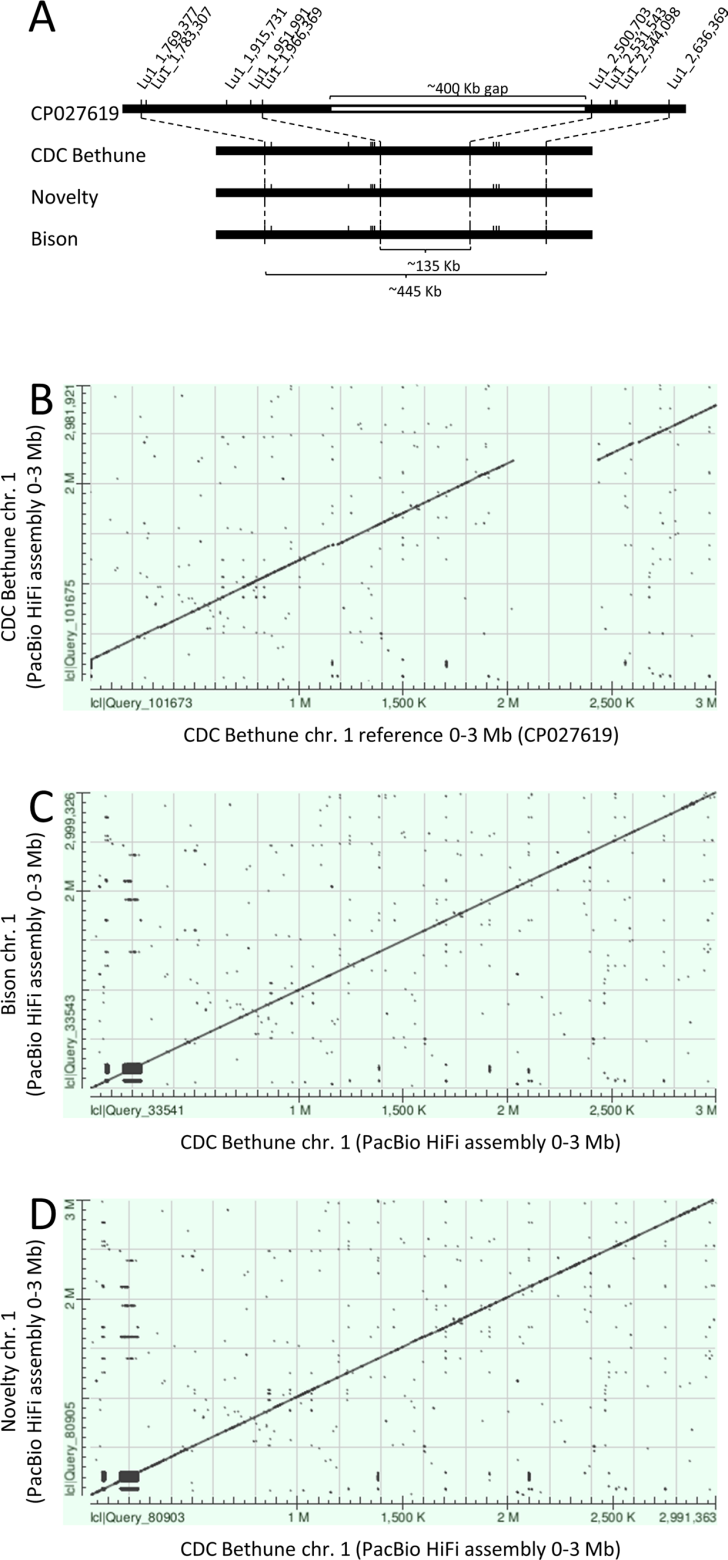
Major Fusarium wilt resistance locus of flax located on chromosome 1 of the ‘CDC Bethune’ reference genome assembly v2.0 (You et al. 2018). **A** Schematic of the region of the ‘CDC Bethune’ chromosome 1 reference sequence (CP027619) spanning 866,993 nucleotides including a long unresolved region of ~ 400 Kb aligned to the ‘CDC Bethune’, ‘Novelty’ and ‘Bison’ assemblies obtained using PacBio HiFi long reads showing the continuity of the sequence without gaps. **B** Dot plot of the alignment between the ‘CDC Bethune’ chromosome 1 reference sequence (CP027619; *x*-axis) and the ‘CDC Bethune’ PacBio HiFi long-read assembly (*y*-axis) illustrating that the predicted unresolved sequence gap of the reference sequence was an artifact. **C** Dot plots of PacBio HiFi long-read assemblies of ‘Bison’ (*y*-axis) and **D** ‘Novelty’ (*y*-axis) against the PacBio HiFi long-read assembly of ‘CDC Bethune’ (*x*-axis) showing the collinearity of the three genomic sequences at the major Fusarium wilt target loci spanning ~ 445 Kb. The names of the SNP markers indicated on top correspond to their position in CP027619 where the acronym Lu1 stands for *Linum usitatissimum* chromosome 1

The Lu1_1,769,377–2,636,369 interval of the ‘CDC Bethune’ reference genome assembly v2.0 contained an unresolved region of 400,099 bp (Fig. 2A). This gap was resolved by sequencing the genome of ‘CDC Bethune’ with PacBio HiFi long reads from which the de novo assembly produced a single contig spanning the QTL. Alignment between this region of chromosome 1 of the ‘CDC Bethune’ reference genome assembly v2.0 (CP027619) and the de novo long-read assembly of ‘CDC Bethune’ indicated that there was no gap in the QTL (Fig. 2B), thereby narrowing its physical distance to 444,755 bp in ‘CDC Bethune’. The ‘Bison’ and ‘Novelty’ whole genome assemblies with PacBio HiFi long reads confirmed the structural organization, i.e., no gap and good collinearity (Fig. 2C, D) with the QTL interval being of similar sizes at 444,285 and 446,701 bp, respectively. These assemblies also provided the primary nucleotide sequence of the resistant and susceptible genotypes over the full length of the target region for the major Fusarium wilt resistance QTL on chromosome 1.

### Fine-mapping with KASP markers

**Fig. 3:**
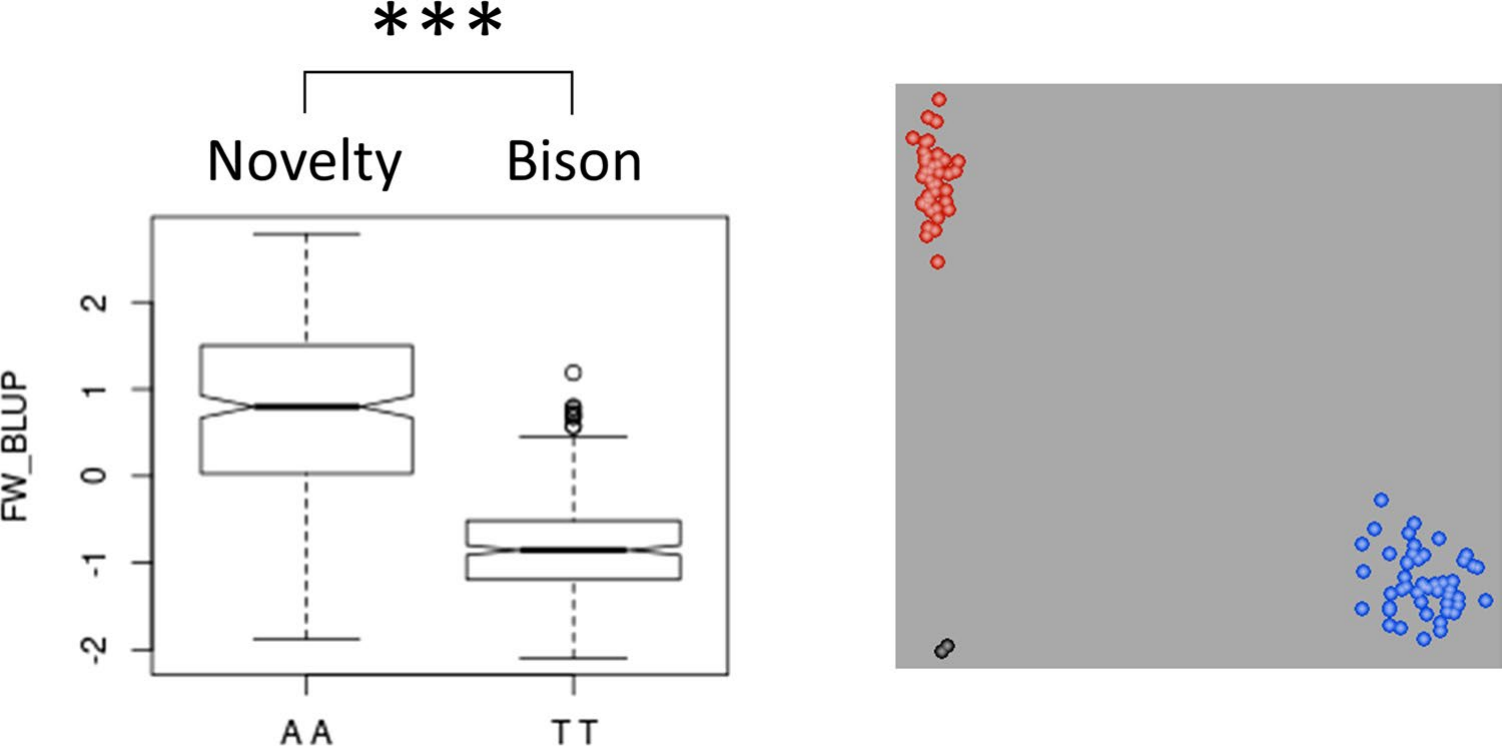
Quantitative trait nucleotide (QTN) Lu1_1,966,369 for Fusarium wilt resistance in flax. Box plot illustrating allelic significance in the ‘Bison’/’Novelty’ recombinant inbred line (RIL) population (left), and KASP assay of the Lu1_1,966,369 marker on a subset of 94 lines (right). *** *P* value < 0.001

**Fig. 4:**
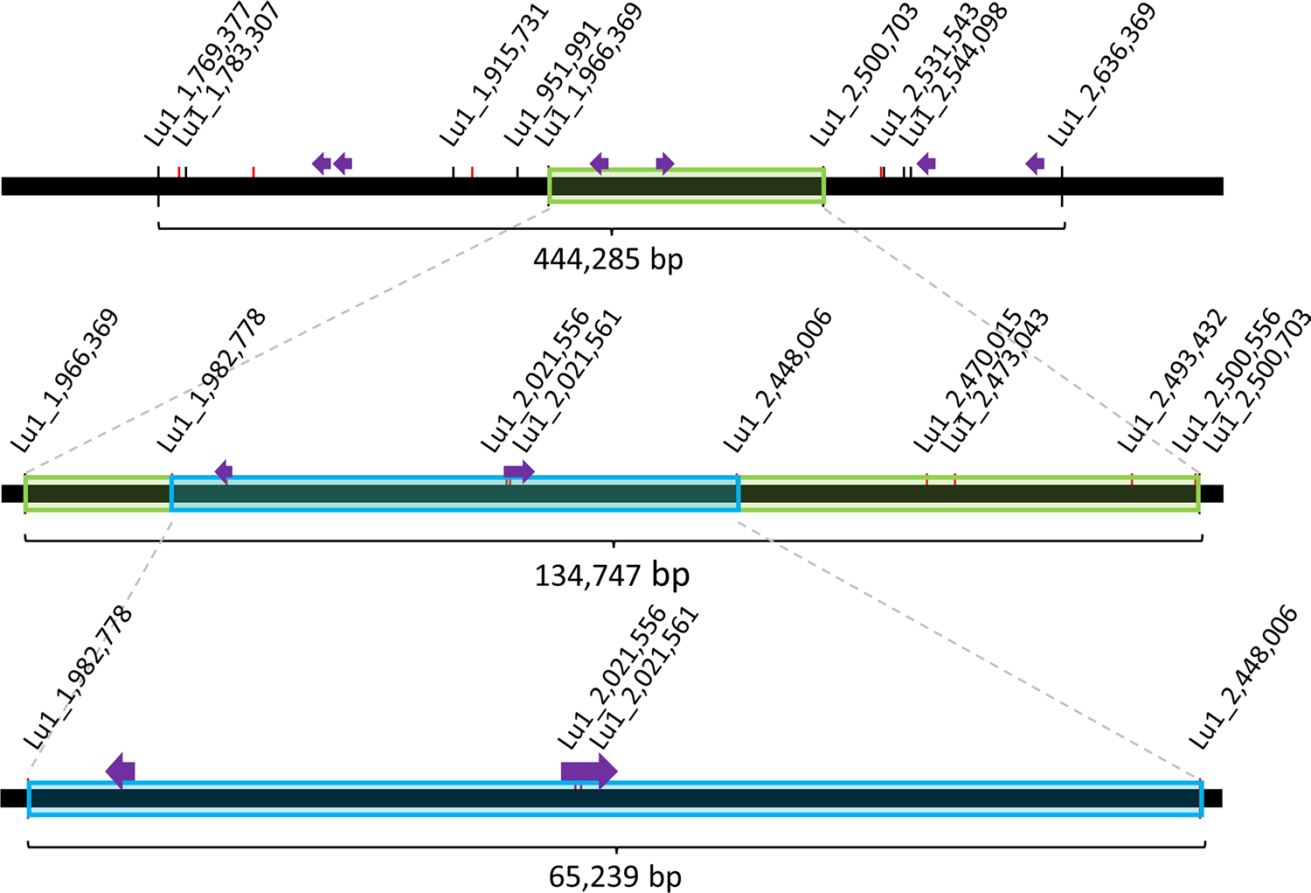
Fine-mapping of the major quantitative trait locus (QTL) for Fusarium wilt resistance located on flax chromosome 1. The top panel shows a ~ 445 Kb interval in ‘Bison’ that harbored 110 genes including six candidate genes for disease resistance (purple arrows). The bottom panel illustrates the ~ 135 Kb interval of the Fusarium wilt QTL. Annotation of this region indicates 39 genes, of which two (purple arrows) have been identified as Fusarium wilt candidate genes based on motifs and predicted functions. Markers are indicated by their base position in the ‘CDC Bethune’ reference assembly v2.0 accession CP027619. SNP markers are indicated by black tick marks while Kompetitive allele-specific PCR (KASP) markers are red (Supplementary Table S4)

Forty-four BN RILs had a recombination between SNP marker Lu1_1,769,377 and Lu1_2,636,369 but six had some residual heterozygosity; hence, a set of 38 fixed recombinants was used for fine-mapping. A first set of seven KASP markers designed within the interval was screened on the BN population to fine-map the QTL (Supplementary Table S4). All markers were robust and displayed significant allelic effects (Fig. 3). Resolution of these KASP markers on the recombinant lines of the BN population located the FW causal genetic feature between Lu1_1,966,369 and Lu1_2,500,703, thereby narrowing the interval to only ~ 135 Kb (Fig. 4). This smaller target region contained 39 genes and comprised candidate genes *Lus10025882* and *Lus10025891*. Within this region, a total 17 BN recombinants were identified. Comparison of the genomic sequence of ‘Bison’ and ‘Novelty’ indicated SNPs and/or indels in 11/39 genes including one SNP and one indel in candidate gene *Lus10025891* but none in *Lus10025882*. An additional eight, all intragenic, KASP markers were designed to span the ~ 135-Kb interval. Their resolution on the recombinant BN RILs fine-mapped the FW resistance QTL to a ~ 65-Kb interval comprising 20 genes, including the two candidates *Lus10025882* and *Lus10025891*.

## Discussion

Fusarium wilt, caused by a soil-borne fungus *Fusarium oxysporum* f. sp. *lini*, is a devastating disease in flax because it persists in the soil for decades and, while mild infection can result in some yield losses due to yellowing and browning, heavy infection can lead to complete crop loss consequent to severe wilting (Booker 2019). Here, we performed a QTL analysis for Fusarium wilt resistance in flax using a population of more than 700 RILs derived from a cross between wilt resistant ‘Bison’ and wilt susceptible ‘Novelty’ using phenotypic data collected from five site-years and genome-wide SNP data. In this study, we employed one recombination-based model, ICIM-ADD in IciMapping, and two non-recombination-based models, namely 3VmrMLM and RTM-GWAS for QTL analysis. These three models identified 24 QTLs, 18 QTNs and 108 LD blocks, respectively, resulting in 129 unique Fusarium wilt resistance loci. Among these, 16 were consistently identified and validated by at least two of the models. Notably, the major QTL on chromosome 1 was consistently detected by all three models. Statistical models for QTL mapping have strengths and limitations. The simultaneous utilization of multiple models is a prudent and effective strategy that leverage their respective strengths, address potential weaknesses and permits cross-validation (Jia et al. 2022; Mueen Alam et al. 2019; Wen et al. 2018).

Considering the accuracy and read length advantages of the PacBio HiFi long-read sequencing technology (https://www.pacb.com/technology/hifi-sequencing/) and the relatively small size of the flax genome (You et al. 2018), obtaining whole genome assemblies of ‘CDC Bethune’, ‘Bison’ and ‘Novelty’ was relatively straight forward and inexpensive. The resulting assemblies, not only corrected errors, e.g., resolving the ~ 400 Kb gap error of the ‘CDC Bethune’ reference genome assembly v2.0 (GeneBank accession CP027619), but also provided complete sequences of the target QTL of both parents, which was paramount to obtaining complete and accurate annotation and facilitating KASP marker design for fine-mapping.

Recently, Kanapin et al. (2021) performed a GWAS for Fusarium wilt resistance in flax using 297 diverse accessions. They identified a major Fusarium wilt resistance locus comprising ten quantitative trait nucleotides (QTNs) spanning a 640-Kb region from position Lu1_1,213,418 to 1,854,337 on chromosome 1 (CP027619). Annotation of the QTL led them to hypothesize *Lus10025717*, a KIP1-like protein and *Lus10025852*, a nucleotide-binding site leucine-rich repeat (NBS-LRR) protein as candidates conferring Fusarium wilt resistance. Using the ‘Bison’/’Novelty’ RIL population, we also identified a major QTL for Fusarium wilt on chromosome 1 comprising 12 SNPs that spanned a 444-Kb region (Lu1_1,769,377–2,636,369) overlapping with that of Kanapin et al. (2021) over ~ 85 Kb (Lu1_1,769,377–1,854,337). Fine-mapping of the locus using KASP markers narrowed the QTL down to ~ 65 Kb between Lu1_1,982,778 and Lu1_2,448,066, which fell outside of the overlapping region; hence, excluding their proposed candidate genes as the possible causal genes for Fusarium wilt resistance in ‘Bison’.

While studying the genome-wide resistance gene analogs in radish (*Raphanus sativus*) to investigate Fusarium wilt resistance candidate genes, Yu et al. (2018) stated that the multitude of classes of Fusarium wilt resistance genes was indicative of the complexity of the infection process. Indeed, the few Fusarium wilt resistance genes isolated to date from tomato (*S. lycopersicum*) and *Arabidopsis* belonged to several classes. In tomato, I-2 is a coiled-coil (CC) NBS-LRR protein (Ori et al. 1997; Simons et al. 1998). Tomato I-3 (Catanzariti et al. 2015) and *Arabidopsis* RFO3 (Cole and Diener 2013) are S-lectin receptor-like kinases (SRLKs). RFO2 in *Arabidopsis* (Shen and Diener 2013) and I-7 (Gonzalez-Cendales et al. 2016) and I (Catanzariti et al. 2017) in tomato are LRR receptor-like protein (RLP). Finally, RFO1 in *Arabidopsis* is a cell-wall-associated RLK (Diener and Ausubel 2005).

The 15 KASP markers designed within the initial ~ 444-Kb QTL and resolved on the fixed recombinant lines permitted the fine-mapping of the QTL to an approximately 69-Kb region that contained only 20 protein-coding genes, including the candidates *Lus10025882* and *Lus10025891*. The former is predicted to encode a serine-threonine protein kinase while the latter is predicted to encode a *G*-type SRLK. Arguments to suggest *Lus10025891* as the primary candidate to confer Fusarium wilt resistance in flax cultivar ‘Bison’ are two-fold. First, the coding sequence of the resistant cultivar ‘Bison’ differs from that of the susceptible cultivar ‘Novelty’ by a 6-bp indel resulting in two fewer leucine residues in ‘Bison’, located close to a G/A SNP responsible for a glutamine (‘Novelty’) to arginine (‘Bison’) substitution while the coding sequence of *Lus10025882* in ‘Bison’ and ‘Novelty’ was identical. Second, two Fusarium wilt resistance proteins, namely I-3 (Catanzariti et al. 2015) in tomato and RFO3 (Cole and Diener 2013) in *Arabidopsis*, are also both *G*-type SRLKs. While these latter two SRLKs specifically confer resistance to the *Fusarium oxysporum* fungal pathogen, SRLKs providing resistance to a range of pathogens have been isolated. Pi-d2, conferring resistance to the rice blast fungus *Magnaporthe grisea*, was the first RLK with a bulb-type mannose specific binding lectin domain identified as a plant disease resistance protein (Chen et al. 2006). Since then, SRLKs involved in a number of host–pathogen systems have been cloned, e.g., the Valsa canker (*Valsa mali*) MdSRLK3 in apple (Han et al. 2022), the phytoplasma Mu-GsSRK in mulberry (Liu et al. 2021) and the bacterial blight (*Xanthomonas oryzae* pv. *oryzae*) OsSPL26 in rice (Shang et al. 2022).

In the last few years, the roles of sensor and helper proteins in effector-triggered immunity in plants have become clearer (van Wersch et al. 2020). The helper proteins identified to date all belong to the NBS-LRR class (Contreras et al. 2023a, b, c; Gabriels et al. 2006; Grant et al. 2003; Peart et al. 2005; Sun et al. 2021). It is therefore unlikely that *Lus10025882* acts as a helper in this pathosystem because it encodes a receptor-like kinase whose primary function is signaling rather than sensing, contrary to the SRLK encoded by *Lus10025891* which contains both an extracellular sensor and intracellular mediator domains (Sun et al. 2020).

Future research will involve validation of the primary Fusarium wilt resistance gene in flax *Lus10025891* through genome editing, complementation and/or mutant analyses. Regardless of the outcome, breeding programs can apply any of the markers developed herein for marker-assisted selection to fix this major Fusarium wilt resistance. Genetic resistance is a major integrated pest management strategy to counteract Fusarium wilt and marker-assisted selection for major genes expedites breeding and facilitates pyramiding (Chitwood-Brown et al. 2021).

### Supplementary Information

Below is the link to the electronic supplementary material.

Supplementary file1 (DOCX 230 KB)

## Data Availability

All data are provided within the manuscript and its Supplementary Materials.
