# Supplementary material for: Fine-mapping of a major locus for Fusarium wilt resistance in flax (Linum usitatissimum L.)
Source: Theor Appl Genet. 2024 Jan 21;137(1):27. doi: 10.1007/s00122-023-04528-2 (PMC10800302; doi:10.1007/s00122-023-04528-2)
Supplement: Supplementary file 1 — Supplementary file1 (DOCX 230 KB) [file 122_2023_4528_MOESM1_ESM.docx]

**Supplementary Materials**

**Fine-mapping of a major locus for Fusarium wilt resistance in flax (*Linum usitatissimum* L.)**

Cloutier S^1^, Edwards T^1^, Zheng C^1^, Booker HM^2,4^, Islam T^2^, Nabetani K^2^, Kutcher HR^2^, Molina O^3^, You FM^1^

^1^ Ottawa Research and Development Centre, Agriculture and Agri-Food Canada, 960 Carling Avenue, Ottawa, ON, Canada K1A 0C6

^2^ Crop Development Centre, University of Saskatchewan, 51 Campus Drive, Saskatoon, SK, Canada S7N 5A8

^3^ Morden Research and Development Centre, Agriculture and Agri-Food Canada, 101 Route 100, Morden, MB, Canada R6M 1Y5

^4^ Current address: Department of Plant Agriculture, Ontario Agricultural College, University of Guelph, 50 Stone Road E, Guelph, ON, Canada N1G 2W1


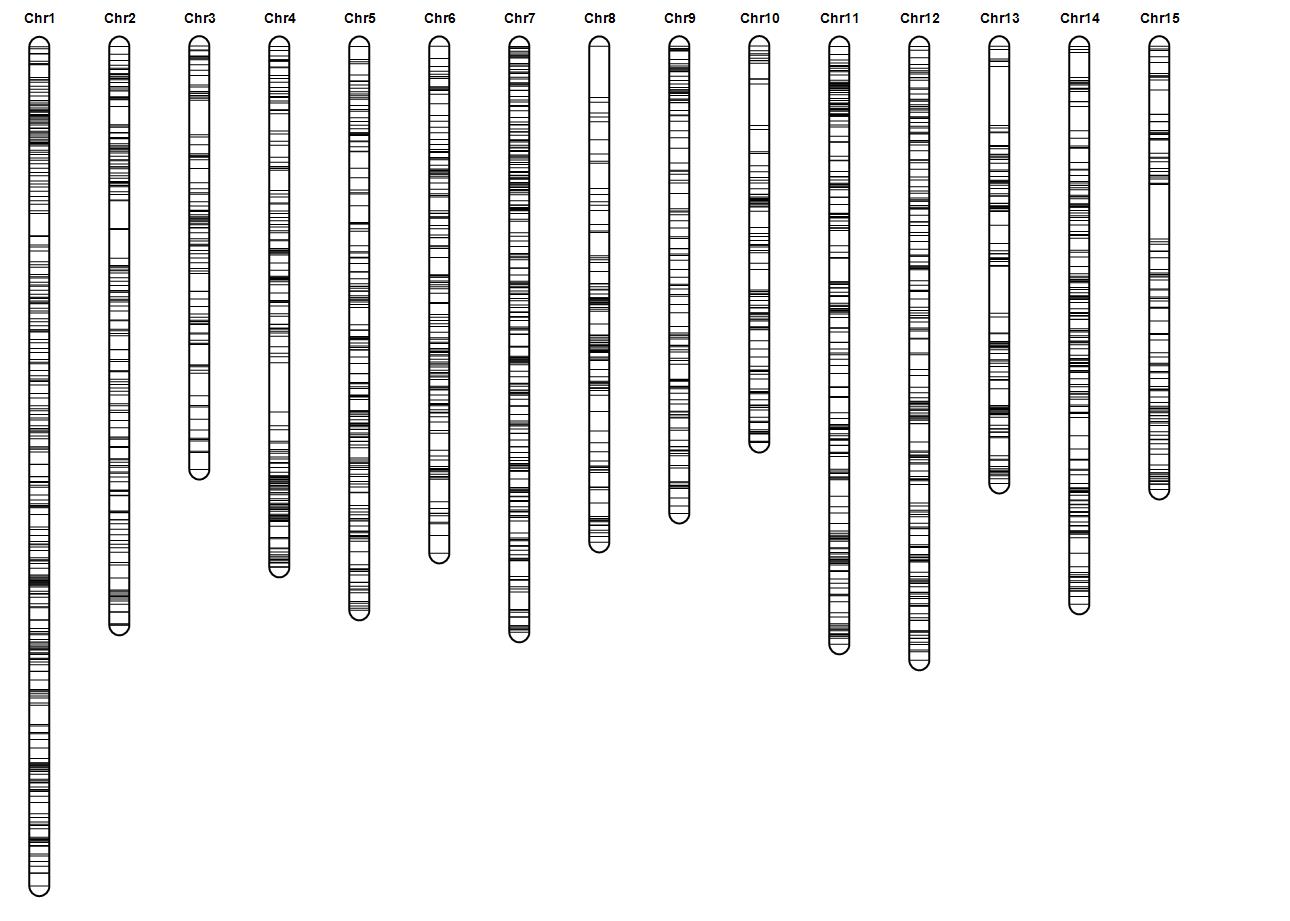


Fig. S1 Genetic map of a 703 recombinant inbred line (RIL) population from the cross ‘Bison’/’Novelty’. The 2374 SNPs spanning all 15 chromosomes cover 4153 cM.


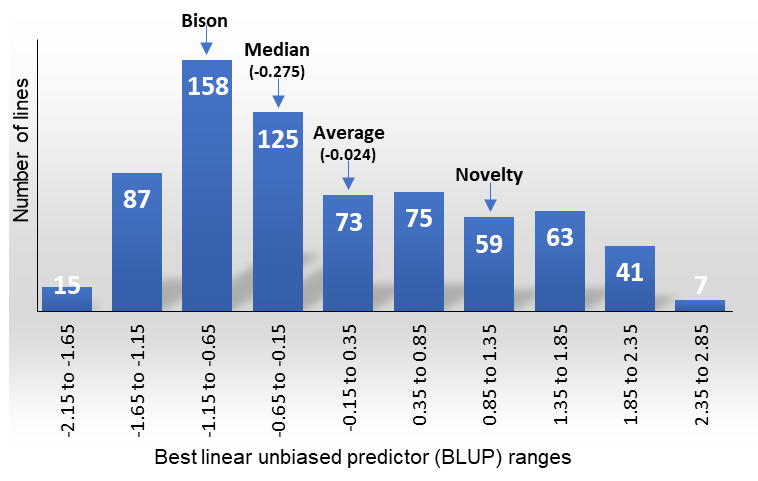


Fig. S2 Frequency distribution of best linear unbiased predictor (BLUP) of Fusarium wilt severity scored on a scale of 0 (healthy and vigorous) to 9 (severely wilted or dead) of the 703 ‘Bison’/’Novelty’ recombinant inbred line (RIL) population showing transgressive segregants at both ends.

Table S1 Single nucleotide polymorphism (SNP) marker distribution on the 15 chromosomes of the flax reference sequence assembly of ‘CDC Bethune’ (You et al. 2018).

| **Accession** | **Chromosome** | **Genetic map** | |
| --- | --- | --- | --- |
|  |  | **No. SNPs** | **Distance (cM)** |
| CP027619 | 1 | 282 | 429 |
| CP027626 | 2 | 161 | 299 |
| CP027627 | 3 | 96 | 221 |
| CP027628 | 4 | 161 | 270 |
| CP027629 | 5 | 172 | 291 |
| CP027630 | 6 | 136 | 263 |
| CP027631 | 7 | 243 | 302 |
| CP027632 | 8 | 107 | 257 |
| CP027633 | 9 | 125 | 243 |
| CP027620 | 10 | 102 | 207 |
| CP027621 | 11 | 199 | 308 |
| CP027622 | 12 | 190 | 316 |
| CP027623 | 13 | 115 | 228 |
| CP027624 | 14 | 178 | 288 |
| CP027625 | 15 | 107 | 231 |
| **Total** |  | **2374** | **4153** |

Table S2 All quantitative trait loci (QTLs), quantitative trait nucleotides (QTNs) and linkage disequilibrium (LD) blocks associated with Fusarium wilt severity in the ‘Bison’/’Novelty’ recombinant inbred line (RIL) population for five site-years, the mean of all site-years and BLUP values as detected by models implemented in IciMapping, IIIVmrMLM and RTM-GWAS. Chromosome, start and end positions (bp) or genetic position (cM), model, dataset, marker, model-specific statistics (LOD or *P* value) and percent of the variance explained (*R^2^*) are listed.

| **QTL No.^a^** | **Statistical model** | **QTL/QTN/LD block No. by model^b^** | **Dataset** | **Chr.** | **Position (cM)** | **Start position (bp)** | **End position (bp)** | **Left marker** | **Right marker** | **LOD^c^** | ***P* value** | **PVE or *R^2^* (%)^d^** |
| --- | --- | --- | --- | --- | --- | --- | --- | --- | --- | --- | --- | --- |
| 1 | RTM-GWAS | 1 | FW-SK2019 | 1 |  | 449771 | 449771 | Lu1_449771 |  |  |  | 1.09 |
| 2 | RTM-GWAS | 2 | FW-SK2019 | 1 |  | 766705 | 766834 | Lu1_LDB_766705_766834 |  |  |  | 1.47 |
| 2 | RTM-GWAS | 2 | FW_SK2021 | 1 |  | 766705 | 766834 | Lu1_LDB_766705_766834 |  |  |  | 0.75 |
| 3 | RTM-GWAS | 3 | FW-SK2019 | 1 |  | 1299854 | 1490954 | Lu1_LDB_1299854_1490954 |  |  |  | 2.88 |
| 4 | RTM-GWAS | 4 | FW-MN2021 | 1 |  | 1523594 | 1711276 | Lu1_LDB_1523594_1711276 |  |  |  | 9.62 |
| 5 | RTM-GWAS | 5 | FW-SK2020 | 1 |  | 1769377 | 1966369 | Lu1_LDB_1769377_1966369 |  |  |  | 23.85 |
| 5 | RTM-GWAS | 5 | FW_MN2019 | 1 |  | 1769377 | 1966369 | Lu1_LDB_1769377_1966369 |  |  |  | 33.01 |
| 5 | RTM-GWAS | 5 | FW_SK2021 | 1 |  | 1769377 | 1966369 | Lu1_LDB_1769377_1966369 |  |  |  | 41.62 |
| 5 | RTM-GWAS | 5 | FW_mean | 1 |  | 1769377 | 1966369 | Lu1_LDB_1769377_1966369 |  |  |  | 40.66 |
| 5 | RTM-GWAS | 5 | FW_BLUP | 1 |  | 1769377 | 1966369 | Lu1_LDB_1769377_1966369 |  |  |  | 40.18 |
| 6 | ICIM-ADD | 1 | FW-SK2020 | 1 | 51 | 1966369 | 2500703 | Lu1_1966369 | Lu1_2500703 | 57.9286 |  | 25.66 |
| 6 | ICIM-ADD | 1 | FW-SK2019 | 1 | 51 | 1966369 | 2500703 | Lu1_1966369 | Lu1_2500703 | 53.2342 |  | 26.59 |
| 6 | ICIM-ADD | 1 | FW_MN2019 | 1 | 51 | 1966369 | 2500703 | Lu1_1966369 | Lu1_2500703 | 105.4395 |  | 41.30 |
| 6 | ICIM-ADD | 1 | FW_SK2021 | 1 | 51 | 1966369 | 2500703 | Lu1_1966369 | Lu1_2500703 | 57.4147 |  | 31.66 |
| 6 | ICIM-ADD | 1 | FW-MN2021 | 1 | 51 | 1966369 | 2500703 | Lu1_1966369 | Lu1_2500703 | 35.4771 |  | 22.63 |
| 6 | ICIM-ADD | 1 | FW_mean | 1 | 51 | 1966369 | 2500703 | Lu1_1966369 | Lu1_2500703 | 143.0472 |  | 47.99 |
| 6 | ICIM-ADD | 1 | FW_BLUP | 1 | 52 | 1966369 | 2500703 | Lu1_1966369 | Lu1_2500703 | 138.7713 |  | 53.84 |
| 6 | 3VmrMLM | 1 | FW-SK2020 | 1 |  | 1966369 |  | Lu1_1966369 |  |  | 0 | 22.41 |
| 6 | 3VmrMLM | 1 | FW-SK2019 | 1 |  | 1966369 |  | Lu1_1966369 |  |  | 0 | 14.23 |
| 6 | 3VmrMLM | 1 | FW_MN2019 | 1 |  | 1966369 |  | Lu1_1966369 |  |  | 0 | 31.45 |
| 6 | 3VmrMLM | 1 | FW_SK2021 | 1 |  | 1966369 |  | Lu1_1966369 |  |  | 0 | 22.90 |
| 6 | 3VmrMLM | 1 | FW-MN2021 | 1 |  | 1966369 |  | Lu1_1966369 |  |  | 0 | 14.25 |
| 6 | 3VmrMLM | 1 | FW_mean | 1 |  | 1966369 |  | Lu1_1966369 |  |  | 0 | 20.11 |
| 6 | 3VmrMLM | 1 | FW_BLUP | 1 |  | 1966369 |  | Lu1_1966369 |  |  | 0 | 20.31 |
| 6 | 3VmrMLM | 2 | FW_mean | 1 |  | 2500703 |  | Lu1_2500703 |  |  | 0 | 4.74 |
| 6 | 3VmrMLM | 2 | FW_BLUP | 1 |  | 2500703 |  | Lu1_2500703 |  |  | 0 | 4.43 |
| 6 | RTM-GWAS | 5 | FW-SK2019 | 1 |  | 2500703 | 2636369 | Lu1_LDB_2500703_2636369 |  |  |  | 17.82 |
| 6 | RTM-GWAS | 5 | FW_MN2019 | 1 |  | 2500703 | 2636369 | Lu1_LDB_2500703_2636369 |  |  |  | 1.34 |
| 6 | RTM-GWAS | 5 | FW_SK2021 | 1 |  | 2500703 | 2636369 | Lu1_LDB_2500703_2636369 |  |  |  | 4.82 |
| 6 | RTM-GWAS | 5 | FW_mean | 1 |  | 2500703 | 2636369 | Lu1_LDB_2500703_2636369 |  |  |  | 1.86 |
| 6 | RTM-GWAS | 5 | FW_BLUP | 1 |  | 2500703 | 2636369 | Lu1_LDB_2500703_2636369 |  |  |  | 1.60 |
| 7 | ICIM-ADD | 2 | FW_SK2021 | 1 | 59 | 2636369 | 2721187 | Lu1_2636369 | Lu1_2721187 | 34.012 |  | 17.37 |
| 7 | RTM-GWAS | 6 | FW_MN2019 | 1 |  | 2653746 | 2653746 | Lu1_2653746 |  |  |  | 1.04 |
| 7 | 3VmrMLM | 3 | FW_SK2021 | 1 |  | 2721187 |  | Lu1_2721187 |  |  | 0 | 6.33 |
| 8 | RTM-GWAS | 7 | FW-MN2021 | 1 |  | 2894739 | 2894739 | Lu1_2894739 |  |  |  | 1.25 |
| 9 | RTM-GWAS | 8 | FW_MN2019 | 1 |  | 3108565 | 3108565 | Lu1_3108565 |  |  |  | 0.40 |
| 9 | RTM-GWAS | 8 | FW_mean | 1 |  | 3108565 | 3108565 | Lu1_3108565 |  |  |  | 1.46 |
| 9 | RTM-GWAS | 8 | FW_BLUP | 1 |  | 3108565 | 3108565 | Lu1_3108565 |  |  |  | 1.22 |
| 10 | RTM-GWAS | 9 | FW_MN2019 | 1 |  | 3377481 | 3377502 | Lu1_LDB_3377481_3377502 |  |  |  | 1.36 |
| 10 | RTM-GWAS | 9 | FW_mean | 1 |  | 3377481 | 3377502 | Lu1_LDB_3377481_3377502 |  |  |  | 0.75 |
| 10 | RTM-GWAS | 9 | FW_BLUP | 1 |  | 3377481 | 3377502 | Lu1_LDB_3377481_3377502 |  |  |  | 0.86 |
| 11 | RTM-GWAS | 10 | FW_SK2021 | 1 |  | 3627256 | 3627256 | Lu1_3627256 |  |  |  | 0.67 |
| 11 | RTM-GWAS | 10 | FW_mean | 1 |  | 3627256 | 3627256 | Lu1_3627256 |  |  |  | 0.34 |
| 11 | RTM-GWAS | 10 | FW_BLUP | 1 |  | 3627256 | 3627256 | Lu1_3627256 |  |  |  | 0.29 |
| 12 | RTM-GWAS | 11 | FW-MN2021 | 1 |  | 5327748 | 5327748 | Lu1_5327748 |  |  |  | 3.46 |
| 12 | RTM-GWAS | 11 | FW_mean | 1 |  | 5327748 | 5327748 | Lu1_5327748 |  |  |  | 0.45 |
| 12 | RTM-GWAS | 11 | FW_BLUP | 1 |  | 5327748 | 5327748 | Lu1_5327748 |  |  |  | 0.30 |
| 13 | ICIM-ADD | 3 | FW_MN2019 | 1 | 156 | 6229534 | 6295779 | Lu1_6229534 | Lu1_6295779 | 4.0815 |  | 1.13 |
| 13 | RTM-GWAS | 12 | FW_BLUP | 1 |  | 6295779 | 6295779 | Lu1_6295779 |  |  |  | 0.71 |
| 14 | RTM-GWAS | 13 | FW_MN2019 | 1 |  | 6405514 | 6417784 | Lu1_LDB_6405514_6417784 |  |  |  | 1.33 |
| 15 | ICIM-ADD | 4 | FW-SK2019 | 1 | 160 | 6624843 | 6768701 | Lu1_6624843 | Lu1_6768701 | 5.222 |  | 2.22 |
| 16 | ICIM-ADD | 5 | FW_mean | 1 | 165 | 6777340 | 6726764 | Lu1_6777340 | Lu1_6726764 | 4.1506 |  | 0.88 |
| 16 | ICIM-ADD | 5 | FW_BLUP | 1 | 165 | 6777340 | 6726764 | Lu1_6777340 | Lu1_6726764 | 3.9398 |  | 0.98 |
| 17 | RTM-GWAS | 14 | FW_MN2019 | 1 |  | 7061656 | 7061656 | Lu1_7061656 |  |  |  | 1.08 |
| 17 | RTM-GWAS | 14 | FW_BLUP | 1 |  | 7061656 | 7061656 | Lu1_7061656 |  |  |  | 0.47 |
| 18 | RTM-GWAS | 15 | FW_SK2021 | 1 |  | 7489291 | 7489291 | Lu1_7489291 |  |  |  | 0.85 |
| 18 | RTM-GWAS | 15 | FW_mean | 1 |  | 7489291 | 7489291 | Lu1_7489291 |  |  |  | 0.34 |
| 19 | RTM-GWAS | 16 | FW_SK2021 | 1 |  | 7525993 | 7525993 | Lu1_7525993 |  |  |  | 0.45 |
| 20 | RTM-GWAS | 17 | FW-MN2021 | 1 |  | 11127983 | 11127983 | Lu1_11127983 |  |  |  | 1.26 |
| 21 | RTM-GWAS | 18 | FW-SK2019 | 1 |  | 18842477 | 18842528 | Lu1_LDB_18842477_18842528 |  |  |  | 1.02 |
| 22 | RTM-GWAS | 19 | FW-MN2021 | 1 |  | 27327348 | 27454676 | Lu1_LDB_27327348_27454676 |  |  |  | 0.26 |
| 23 | RTM-GWAS | 20 | FW-SK2019 | 1 |  | 27507551 | 27576434 | Lu1_LDB_27507551_27576434 |  |  |  | 1.15 |
| 23 | RTM-GWAS | 20 | FW_SK2021 | 1 |  | 27507551 | 27576434 | Lu1_LDB_27507551_27576434 |  |  |  | 0.84 |
| 23 | RTM-GWAS | 20 | FW-MN2021 | 1 |  | 27507551 | 27576434 | Lu1_LDB_27507551_27576434 |  |  |  | 3.78 |
| 23 | RTM-GWAS | 20 | FW_mean | 1 |  | 27507551 | 27576434 | Lu1_LDB_27507551_27576434 |  |  |  | 1.22 |
| 23 | RTM-GWAS | 20 | FW_BLUP | 1 |  | 27507551 | 27576434 | Lu1_LDB_27507551_27576434 |  |  |  | 1.47 |
| 24 | RTM-GWAS | 21 | FW-SK2020 | 1 |  | 28791062 | 28791086 | Lu1_LDB_28791062_28791086 |  |  |  | 1.07 |
| 25 | RTM-GWAS | 22 | FW-SK2020 | 2 |  | 2091607 | 2247540 | Lu2_LDB_2091607_2247540 |  |  |  | 2.19 |
| 26 | RTM-GWAS | 23 | FW_MN2019 | 2 |  | 3073863 | 3073863 | Lu2_3073863 |  |  |  | 0.69 |
| 27 | RTM-GWAS | 24 | FW_SK2021 | 2 |  | 3879859 | 3880410 | Lu2_LDB_3879859_3880410 |  |  |  | 1.16 |
| 28 | RTM-GWAS | 25 | FW_MN2019 | 2 |  | 4749239 | 4749239 | Lu2_4749239 |  |  |  | 2.70 |
| 29 | RTM-GWAS | 26 | FW-SK2019 | 2 |  | 17456792 | 17456792 | Lu2_17456792 |  |  |  | 1.59 |
| 29 | RTM-GWAS | 26 | FW_mean | 2 |  | 17456792 | 17456792 | Lu2_17456792 |  |  |  | 0.56 |
| 29 | RTM-GWAS | 26 | FW_BLUP | 2 |  | 17456792 | 17456792 | Lu2_17456792 |  |  |  | 0.94 |
| 30 | RTM-GWAS | 27 | FW_MN2019 | 2 |  | 21530990 | 21530990 | Lu2_21530990 |  |  |  | 1.12 |
| 31 | RTM-GWAS | 28 | FW_MN2019 | 3 |  | 3637352 | 3637371 | Lu3_LDB_3637352_3637371 |  |  |  | 1.49 |
| 32 | ICIM-ADD | 6 | FW_SK2021 | 3 | 67 | 4381469 | 5517776 | Lu3_4381469 | Lu3_5517776 | 5.4184 |  | 2.81 |
| 32 | 3VmrMLM | 4 | FW_SK2021 | 3 |  | 4381469 |  | Lu3_4381469 |  |  | 9.88E-06 | 1.16 |
| 32 | 3VmrMLM | 4 | FW_mean | 3 |  | 4381469 |  | Lu3_4381469 |  |  | 6.5E-08 | 1.50 |
| 32 | 3VmrMLM | 4 | FW_BLUP | 3 |  | 4381469 |  | Lu3_4381469 |  |  | 4.75E-08 | 1.54 |
| 32 | RTM-GWAS | 29 | FW_mean | 3 |  | 4381469 | 4381469 | Lu3_4381469 |  |  |  | 3.00 |
| 32 | RTM-GWAS | 29 | FW_BLUP | 3 |  | 4381469 | 4381469 | Lu3_4381469 |  |  |  | 3.10 |
| 32 | RTM-GWAS | 30 | FW-SK2020 | 3 |  | 5517776 | 5517776 | Lu3_5517776 |  |  |  | 1.21 |
| 32 | RTM-GWAS | 30 | FW_SK2021 | 3 |  | 5517776 | 5517776 | Lu3_5517776 |  |  |  | 1.69 |
| 33 | RTM-GWAS | 31 | FW_MN2019 | 3 |  | 6360473 | 6369922 | Lu3_LDB_6360473_6369922 |  |  |  | 1.24 |
| 34 | RTM-GWAS | 32 | FW_BLUP | 3 |  | 17221818 | 17221818 | Lu3_17221818 |  |  |  | 0.36 |
| 35 | RTM-GWAS | 33 | FW_BLUP | 3 |  | 17593912 | 17593912 | Lu3_17593912 |  |  |  | 0.46 |
| 36 | ICIM-ADD | 7 | FW-SK2019 | 3 | 148 | 19965022 | 22290905 | Lu1_19965022 | Lu3_22290905 | 4.5474 |  | 2.04 |
| 36 | ICIM-ADD | 7 | FW_MN2019 | 3 | 149 | 19965022 | 22290905 | Lu1_19965022 | Lu3_22290905 | 4.9474 |  | 1.42 |
| 37 | ICIM-ADD | 8 | FW_mean | 3 | 150 | 22290905 | 22290944 | Lu3_22290905 | Lu3_22290944 | 4.4842 |  | 0.91 |
| 37 | ICIM-ADD | 8 | FW_BLUP | 3 | 150 | 22290905 | 22290944 | Lu3_22290905 | Lu3_22290944 | 4.5535 |  | 1.09 |
| 37 | RTM-GWAS | 34 | FW-SK2019 | 3 |  | 22290905 | 22290944 | Lu3_LDB_22290905_22290944 |  |  |  | 1.40 |
| 37 | RTM-GWAS | 34 | FW_MN2019 | 3 |  | 22290905 | 22290944 | Lu3_LDB_22290905_22290944 |  |  |  | 4.83 |
| 37 | RTM-GWAS | 34 | FW_mean | 3 |  | 22290905 | 22290944 | Lu3_LDB_22290905_22290944 |  |  |  | 1.68 |
| 37 | RTM-GWAS | 34 | FW_BLUP | 3 |  | 22290905 | 22290944 | Lu3_LDB_22290905_22290944 |  |  |  | 1.61 |
| 37 | 3VmrMLM | 5 | FW_MN2019 | 3 |  | 22290944 |  | Lu3_22290944 |  |  | 0 | 3.66 |
| 38 | RTM-GWAS | 35 | FW_MN2019 | 3 |  | 23375864 | 23375900 | Lu3_LDB_23375864_23375900 |  |  |  | 0.79 |
| 39 | RTM-GWAS | 36 | FW-SK2019 | 4 |  | 12774929 | 12956773 | Lu4_LDB_12774929_12956773 |  |  |  | 3.86 |
| 40 | ICIM-ADD | 9 | FW-SK2019 | 4 | 141 | 13290133 | 13800345 | Lu4_13290133 | Lu4_13800345 | 3.8012 |  | 1.71 |
| 41 | ICIM-ADD | 11 | FW_mean | 4 | 150 | 13571335 | 14284199 | Lu4_13571335 | Lu4_14284199 | 10.7026 |  | 2.22 |
| 41 | ICIM-ADD | 10 | FW_MN2019 | 4 | 144 | 13800341 | 13723558 | Lu4_13800341 | Lu4_13723558 | 4.648 |  | 1.27 |
| 41 | ICIM-ADD | 10 | FW_BLUP | 4 | 144 | 13800341 | 13723558 | Lu4_13800341 | Lu4_13723558 | 6.3822 |  | 1.54 |
| 41 | ICIM-ADD | 10 | FW_mean | 4 | 145 | 13800341 | 13723558 | Lu4_13800341 | Lu4_13723558 | 28.0715 |  | 6.56 |
| 41 | 3VmrMLM | 6 | FW_mean | 4 |  | 13800341 |  | Lu4_13800341 |  |  | 3.36E-07 | 1.34 |
| 41 | 3VmrMLM | 6 | FW_BLUP | 4 |  | 13800341 |  | Lu4_13800341 |  |  | 5.38E-07 | 1.29 |
| 42 | RTM-GWAS | 37 | FW-MN2021 | 4 |  | 14747328 | 14747328 | Lu4_14747328 |  |  |  | 1.78 |
| 43 | RTM-GWAS | 38 | FW_mean | 4 |  | 15038993 | 15038993 | Lu4_15038993 |  |  |  | 0.95 |
| 44 | RTM-GWAS | 38 | FW_BLUP | 4 |  | 15038993 | 15038993 | Lu4_15038993 |  |  |  | 1.12 |
| 45 | RTM-GWAS | 39 | FW_SK2021 | 4 |  | 17152996 | 17153002 | Lu4_LDB_17152996_17153002 |  |  |  | 1.22 |
| 46 | RTM-GWAS | 40 | FW_SK2021 | 4 |  | 17308842 | 17310347 | Lu4_LDB_17308842_17310347 |  |  |  | 1.61 |
| 46 | RTM-GWAS | 40 | FW_mean | 4 |  | 17308842 | 17310347 | Lu4_LDB_17308842_17310347 |  |  |  | 1.53 |
| 46 | RTM-GWAS | 40 | FW_BLUP | 4 |  | 17308842 | 17310347 | Lu4_LDB_17308842_17310347 |  |  |  | 1.40 |
| 47 | RTM-GWAS | 41 | FW_MN2019 | 4 |  | 17347955 | 17348000 | Lu4_LDB_17347955_17348000 |  |  |  | 1.40 |
| 48 | 3VmrMLM | 7 | FW_SK2021 | 4 |  | 17523769 |  | Lu4_17523769 |  |  | 2.52E-06 | 1.54 |
| 49 | 3VmrMLM | 8 | FW-SK2019 | 5 |  | 1125627 |  | Lu5_1125627 |  |  | 3.83E-06 | 1.91 |
| 49 | RTM-GWAS | 42 | FW-SK2019 | 5 |  | 1125627 | 1126421 | Lu5_LDB_1125627_1126421 |  |  |  | 2.60 |
| 50 | RTM-GWAS | 43 | FW-MN2021 | 5 |  | 1753082 | 1864302 | Lu5_LDB_1753082_1864302 |  |  |  | 2.23 |
| 51 | RTM-GWAS | 44 | FW_mean | 5 |  | 1934220 | 1934381 | Lu5_LDB_1934220_1934381 |  |  |  | 0.87 |
| 52 | 3VmrMLM | 9 | FW-SK2019 | 5 |  | 4644765 |  | Lu5_4644765 |  |  | 7.76E-07 | 2.21 |
| 52 | RTM-GWAS | 45 | FW-SK2019 | 5 |  | 4644765 | 4644765 | Lu5_4644765 |  |  |  | 2.69 |
| 52 | RTM-GWAS | 45 | FW_BLUP | 5 |  | 4644765 | 4644765 | Lu5_4644765 |  |  |  | 0.50 |
| 53 | RTM-GWAS | 46 | FW_BLUP | 5 |  | 12029402 | 12029402 | Lu5_12029402 |  |  |  | 0.35 |
| 54 | RTM-GWAS | 47 | FW_SK2021 | 5 |  | 12455201 | 12466000 | Lu5_LDB_12455201_12466000 |  |  |  | 1.06 |
| 55 | RTM-GWAS | 48 | FW-SK2019 | 5 |  | 13917283 | 14007105 | Lu5_LDB_13917283_14007105 |  |  |  | 2.54 |
| 56 | RTM-GWAS | 49 | FW_mean | 5 |  | 15477464 | 15566313 | Lu5_LDB_15477464_15566313 |  |  |  | 1.07 |
| 56 | RTM-GWAS | 49 | FW_BLUP | 5 |  | 15477464 | 15566313 | Lu5_LDB_15477464_15566313 |  |  |  | 0.90 |
| 56 | RTM-GWAS | 50 | FW-MN2021 | 5 |  | 15831281 | 16020899 | Lu5_LDB_15831281_16020899 |  |  |  | 1.14 |
| 57 | RTM-GWAS | 51 | FW-SK2020 | 5 |  | 17408530 | 17416522 | Lu5_LDB_17408530_17416522 |  |  |  | 1.34 |
| 57 | RTM-GWAS | 51 | FW_MN2019 | 5 |  | 17408530 | 17416522 | Lu5_LDB_17408530_17416522 |  |  |  | 2.71 |
| 57 | RTM-GWAS | 51 | FW_mean | 5 |  | 17408530 | 17416522 | Lu5_LDB_17408530_17416522 |  |  |  | 2.48 |
| 57 | RTM-GWAS | 51 | FW_BLUP | 5 |  | 17408530 | 17416522 | Lu5_LDB_17408530_17416522 |  |  |  | 2.31 |
| 58 | RTM-GWAS | 52 | FW_mean | 6 |  | 7544173 | 7553864 | Lu6_LDB_7544173_7553864 |  |  |  | 0.40 |
| 58 | RTM-GWAS | 52 | FW_BLUP | 6 |  | 7544173 | 7553864 | Lu6_LDB_7544173_7553864 |  |  |  | 0.60 |
| 59 | RTM-GWAS | 53 | FW_mean | 6 |  | 12400685 | 12400685 | Lu6_12400685 |  |  |  | 0.68 |
| 59 | RTM-GWAS | 53 | FW_BLUP | 6 |  | 12400685 | 12400685 | Lu6_12400685 |  |  |  | 0.44 |
| 60 | ICIM-ADD | 12 | FW_MN2019 | 6 | 141 | 12801500 | 12679391 | Lu6_12801500 | Lu6_12679391 | 6.4178 |  | 1.79 |
| 61 | ICIM-ADD | 12 | FW-SK2020 | 6 | 148 | 12806216 | 13114880 | Lu6_12806216 | Lu6_13114880 | 13.4046 |  | 5.05 |
| 61 | RTM-GWAS | 54 | FW_MN2019 | 6 |  | 13114880 | 13114880 | Lu6_13114880 |  |  |  | 2.32 |
| 61 | RTM-GWAS | 54 | FW_mean | 6 |  | 13114880 | 13114880 | Lu6_13114880 |  |  |  | 1.14 |
| 61 | RTM-GWAS | 54 | FW_BLUP | 6 |  | 13114880 | 13114880 | Lu6_13114880 |  |  |  | 1.64 |
| 62 | RTM-GWAS | 55 | FW-MN2021 | 6 |  | 13241662 | 13241662 | Lu6_13241662 |  |  |  | 2.86 |
| 63 | RTM-GWAS | 56 | FW-SK2019 | 6 |  | 13639951 | 13718973 | Lu6_LDB_13639951_13718973 |  |  |  | 2.60 |
| 64 | ICIM-ADD | 13 | FW-SK2020 | 6 | 163 | 13833145 | 13856270 | Lu6_13833145 | Lu6_13856270 | 7.9658 |  | 3.02 |
| 65 | ICIM-ADD | 14 | FW-SK2019 | 6 | 164 | 13856270 | 13957633 | Lu6_13856270 | Lu6_13957633 | 5.6162 |  | 2.39 |
| 66 | RTM-GWAS | 57 | FW-SK2020 | 6 |  | 15634509 | 15638707 | Lu6_LDB_15634509_15638707 |  |  |  | 1.49 |
| 66 | RTM-GWAS | 57 | FW-SK2019 | 6 |  | 15634509 | 15638707 | Lu6_LDB_15634509_15638707 |  |  |  | 1.35 |
| 66 | RTM-GWAS | 57 | FW-MN2021 | 6 |  | 15634509 | 15638707 | Lu6_LDB_15634509_15638707 |  |  |  | 1.67 |
| 66 | RTM-GWAS | 57 | FW_mean | 6 |  | 15634509 | 15638707 | Lu6_LDB_15634509_15638707 |  |  |  | 1.62 |
| 66 | RTM-GWAS | 57 | FW_BLUP | 6 |  | 15634509 | 15638707 | Lu6_LDB_15634509_15638707 |  |  |  | 1.56 |
| 67 | RTM-GWAS | 58 | FW_MN2019 | 7 |  | 1346858 | 1533205 | Lu7_LDB_1346858_1533205 |  |  |  | 1.21 |
| 68 | RTM-GWAS | 59 | FW-SK2020 | 7 |  | 1985120 | 2071107 | Lu7_LDB_1985120_2071107 |  |  |  | 2.44 |
| 68 | RTM-GWAS | 59 | FW_MN2019 | 7 |  | 1985120 | 2071107 | Lu7_LDB_1985120_2071107 |  |  |  | 1.82 |
| 68 | RTM-GWAS | 59 | FW_mean | 7 |  | 1985120 | 2071107 | Lu7_LDB_1985120_2071107 |  |  |  | 1.09 |
| 68 | RTM-GWAS | 59 | FW_BLUP | 7 |  | 1985120 | 2071107 | Lu7_LDB_1985120_2071107 |  |  |  | 1.11 |
| 69 | RTM-GWAS | 60 | FW_BLUP | 7 |  | 2406815 | 2515634 | Lu7_LDB_2406815_2515634 |  |  |  | 0.42 |
| 70 | RTM-GWAS | 61 | FW_BLUP | 7 |  | 2703901 | 2850949 | Lu7_LDB_2703901_2850949 |  |  |  | 0.40 |
| 71 | RTM-GWAS | 62 | FW-SK2020 | 7 |  | 15993215 | 16011543 | Lu7_LDB_15993215_16011543 |  |  |  | 1.54 |
| 72 | RTM-GWAS | 63 | FW_SK2021 | 7 |  | 16327970 | 16411424 | Lu7_LDB_16327970_16411424 |  |  |  | 0.74 |
| 72 | RTM-GWAS | 63 | FW_mean | 7 |  | 16327970 | 16411424 | Lu7_LDB_16327970_16411424 |  |  |  | 0.65 |
| 72 | RTM-GWAS | 63 | FW_BLUP | 7 |  | 16327970 | 16411424 | Lu7_LDB_16327970_16411424 |  |  |  | 1.09 |
| 73 | RTM-GWAS | 64 | FW-SK2020 | 8 |  | 3065247 | 3065247 | Lu8_3065247 |  |  |  | 0.65 |
| 74 | RTM-GWAS | 65 | FW-MN2021 | 8 |  | 4407094 | 4407094 | Lu8_4407094 |  |  |  | 1.47 |
| 75 | RTM-GWAS | 66 | FW-MN2021 | 8 |  | 4626710 | 4626710 | Lu8_4626710 |  |  |  | 1.42 |
| 76 | ICIM-ADD | 15 | FW_MN2019 | 8 | 150 | 19007240 | 19104645 | Lu8_19007240 | Lu8_19104645 | 15.4373 |  | 4.37 |
| 77 | 3VmrMLM | 10 | FW_MN2019 | 8 |  | 19118386 |  | Lu8_19118386 |  |  | 0 | 4.83 |
| 78 | ICIM-ADD | 18 | FW-SK2019 | 9 | 163 | 1126421 | 1125627 | Lu5_1126421 | Lu5_1125627 | 4.1212 |  | 1.78 |
| 79 | RTM-GWAS | 67 | FW-SK2019 | 9 |  | 3410246 | 3410402 | Lu9_LDB_3410246_3410402 |  |  |  | 0.83 |
| 79 | RTM-GWAS | 67 | FW-MN2021 | 9 |  | 3410246 | 3410402 | Lu9_LDB_3410246_3410402 |  |  |  | 1.24 |
| 79 | RTM-GWAS | 67 | FW_mean | 9 |  | 3410246 | 3410402 | Lu9_LDB_3410246_3410402 |  |  |  | 0.70 |
| 79 | RTM-GWAS | 67 | FW_BLUP | 9 |  | 3410246 | 3410402 | Lu9_LDB_3410246_3410402 |  |  |  | 0.74 |
| 80 | RTM-GWAS | 68 | FW_MN2019 | 9 |  | 6065077 | 6065077 | Lu9_6065077 |  |  |  | 1.19 |
| 81 | RTM-GWAS | 69 | FW_MN2019 | 9 |  | 7314682 | 7314730 | Lu9_LDB_7314682_7314730 |  |  |  | 1.33 |
| 82 | ICIM-ADD | 16 | FW-SK2020 | 9 | 125 | 7314730 | 8411940 | Lu9_7314730 | Lu9_8411940 | 4.0884 |  | 1.55 |
| 82 | 3VmrMLM | 11 | FW-SK2020 | 9 |  | 7314730 |  | Lu9_7314730 |  |  | 4.07E-06 | 2.10 |
| 83 | RTM-GWAS | 70 | FW-SK2020 | 9 |  | 16292196 | 16292196 | Lu9_16292196 |  |  |  | 0.92 |
| 84 | 3VmrMLM | 12 | FW_MN2019 | 9 |  | 17812980 |  | Lu9_17812980 |  |  | 4.3E-10 | 2.43 |
| 85 | ICIM-ADD | 17 | FW_MN2019 | 9 | 154 | 18020087 | 18325581 | Lu9_18020087 | Lu9_18325581 | 9.4601 |  | 2.75 |
| 85 | ICIM-ADD | 17 | FW_mean | 9 | 155 | 18020087 | 18325581 | Lu9_18020087 | Lu9_18325581 | 8.3784 |  | 1.78 |
| 85 | ICIM-ADD | 17 | FW_BLUP | 9 | 155 | 18020087 | 18325581 | Lu9_18020087 | Lu9_18325581 | 7.7392 |  | 1.93 |
| 85 | 3VmrMLM | 13 | FW_mean | 9 |  | 18325581 |  | Lu9_18325581 |  |  | 3.47E-10 | 2.05 |
| 85 | 3VmrMLM | 13 | FW_BLUP | 9 |  | 18325581 |  | Lu9_18325581 |  |  | 5.02E-10 | 2.01 |
| 86 | RTM-GWAS | 71 | FW-SK2020 | 9 |  | 18680722 | 18680722 | Lu9_18680722 |  |  |  | 1.03 |
| 87 | RTM-GWAS | 72 | FW-SK2020 | 10 |  | 3736928 | 3737078 | Lu10_LDB_3736928_3737078 |  |  |  | 2.11 |
| 88 | RTM-GWAS | 73 | FW-MN2021 | 10 |  | 9868326 | 9868625 | Lu10_LDB_9868326_9868625 |  |  |  | 2.24 |
| 89 | RTM-GWAS | 74 | FW_MN2019 | 10 |  | 11058422 | 11137741 | Lu10_LDB_11058422_11137741 |  |  |  | 1.22 |
| 90 | RTM-GWAS | 75 | FW_SK2021 | 10 |  | 13662971 | 13749901 | Lu10_LDB_13662971_13749901 |  |  |  | 0.97 |
| 90 | RTM-GWAS | 75 | FW_mean | 10 |  | 13662971 | 13749901 | Lu10_LDB_13662971_13749901 |  |  |  | 0.90 |
| 90 | RTM-GWAS | 75 | FW_BLUP | 10 |  | 13662971 | 13749901 | Lu10_LDB_13662971_13749901 |  |  |  | 0.75 |
| 91 | RTM-GWAS | 76 | FW-MN2021 | 10 |  | 13988842 | 13988842 | Lu10_13988842 |  |  |  | 2.53 |
| 92 | RTM-GWAS | 77 | FW-MN2021 | 11 |  | 2773062 | 2773079 | Lu11_LDB_2773062_2773079 |  |  |  | 1.46 |
| 93 | RTM-GWAS | 78 | FW_MN2019 | 11 |  | 3686484 | 3686484 | Lu11_3686484 |  |  |  | 1.35 |
| 94 | ICIM-ADD | 19 | FW_MN2019 | 11 | 240 | 17576902 | 17682834 | Lu11_17576902 | Lu11_17682834 | 4.3338 |  | 1.23 |
| 95 | RTM-GWAS | 79 | FW_MN2019 | 12 |  | 28490 | 196181 | Lu12_LDB_28490_196181 |  |  |  | 0.74 |
| 96 | RTM-GWAS | 80 | FW_mean | 12 |  | 889397 | 889450 | Lu12_LDB_889397_889450 |  |  |  | 0.56 |
| 97 | ICIM-ADD | 20 | FW-SK2020 | 12 | 86 | 2382993 | 2399472 | Lu12_2382993 | Lu12_2399472 | 3.5337 |  | 1.36 |
| 98 | ICIM-ADD | 21 | FW_mean | 12 | 145 | 4538390 | 4538511 | Lu12_4538390 | Lu12_4538511 | 3.706 |  | 0.75 |
| 98 | ICIM-ADD | 21 | FW_BLUP | 12 | 145 | 4538390 | 4538511 | Lu12_4538390 | Lu12_4538511 | 3.84 |  | 0.92 |
| 99 | ICIM-ADD | 22 | FW_MN2019 | 12 | 156 | 4578833 | 5067835 | Lu12_4578833 | Lu12_5067835 | 4.1904 |  | 1.31 |
| 100 | RTM-GWAS | 81 | FW_SK2021 | 12 |  | 11601788 | 11716549 | Lu12_LDB_11601788_11716549 |  |  |  | 1.59 |
| 100 | RTM-GWAS | 81 | FW_mean | 12 |  | 11601788 | 11716549 | Lu12_LDB_11601788_11716549 |  |  |  | 0.66 |
| 100 | RTM-GWAS | 81 | FW_BLUP | 12 |  | 11601788 | 11716549 | Lu12_LDB_11601788_11716549 |  |  |  | 0.58 |
| 101 | RTM-GWAS | 82 | FW-SK2020 | 12 |  | 18088587 | 18088587 | Lu12_18088587 |  |  |  | 1.38 |
| 102 | 3VmrMLM | 14 | FW-SK2020 | 12 |  | 18325278 |  | Lu12_18325278 |  |  | 4.25E-05 | 1.46 |
| 103 | ICIM-ADD | 23 | FW_mean | 12 | 242 | 18416660 | 18448659 | Lu12_18416660 | Lu12_18448659 | 5.0572 |  | 1.04 |
| 103 | ICIM-ADD | 23 | FW_BLUP | 12 | 242 | 18416660 | 18448659 | Lu12_18416660 | Lu12_18448659 | 4.5455 |  | 1.09 |
| 104 | 3VmrMLM | 15 | FW_MN2019 | 12 |  | 18448659 |  | Lu12_18448659 |  |  | 1.24E-07 | 1.75 |
| 104 | 3VmrMLM | 15 | FW-MN2021 | 12 |  | 18448659 |  | Lu12_18448659 |  |  | 9.09E-06 | 2.39 |
| 104 | 3VmrMLM | 15 | FW_mean | 12 |  | 18448659 |  | Lu12_18448659 |  |  | 1.71E-10 | 2.14 |
| 104 | 3VmrMLM | 15 | FW_BLUP | 12 |  | 18448659 |  | Lu12_18448659 |  |  | 5.05E-10 | 2.02 |
| 104 | RTM-GWAS | 83 | FW_MN2019 | 12 |  | 18448659 | 18448659 | Lu12_18448659 |  |  |  | 2.07 |
| 104 | RTM-GWAS | 83 | FW-MN2021 | 12 |  | 18448659 | 18448659 | Lu12_18448659 |  |  |  | 3.77 |
| 104 | RTM-GWAS | 83 | FW_mean | 12 |  | 18448659 | 18448659 | Lu12_18448659 |  |  |  | 3.91 |
| 104 | RTM-GWAS | 83 | FW_BLUP | 12 |  | 18448659 | 18448659 | Lu12_18448659 |  |  |  | 3.76 |
| 105 | RTM-GWAS | 84 | FW-SK2020 | 12 |  | 18855826 | 18870803 | Lu12_LDB_18855826_18870803 |  |  |  | 2.52 |
| 105 | RTM-GWAS | 84 | FW_SK2021 | 12 |  | 18855826 | 18870803 | Lu12_LDB_18855826_18870803 |  |  |  | 1.67 |
| 105 | RTM-GWAS | 84 | FW-MN2021 | 12 |  | 18855826 | 18870803 | Lu12_LDB_18855826_18870803 |  |  |  | 6.56 |
| 105 | RTM-GWAS | 84 | FW_mean | 12 |  | 18855826 | 18870803 | Lu12_LDB_18855826_18870803 |  |  |  | 0.99 |
| 105 | RTM-GWAS | 84 | FW_BLUP | 12 |  | 18855826 | 18870803 | Lu12_LDB_18855826_18870803 |  |  |  | 0.80 |
| 106 | RTM-GWAS | 85 | FW_SK2021 | 12 |  | 20063972 | 20100027 | Lu12_LDB_20063972_20100027 |  |  |  | 0.83 |
| 107 | RTM-GWAS | 86 | FW-SK2020 | 12 |  | 20249161 | 20249161 | Lu12_20249161 |  |  |  | 1.32 |
| 108 | RTM-GWAS | 87 | FW_SK2021 | 12 |  | 20716410 | 20716410 | Lu12_20716410 |  |  |  | 0.45 |
| 109 | RTM-GWAS | 88 | FW_MN2019 | 13 |  | 1769062 | 1888694 | Lu13_LDB_1769062_1888694 |  |  |  | 1.06 |
| 110 | RTM-GWAS | 89 | FW_MN2019 | 13 |  | 3493579 | 3493579 | Lu13_3493579 |  |  |  | 1.36 |
| 110 | RTM-GWAS | 90 | FW-SK2019 | 13 |  | 5538460 | 5538510 | Lu13_LDB_5538460_5538510 |  |  |  | 1.26 |
| 111 | ICIM-ADD | 24 | FW_MN2019 | 13 | 18 | 5544664 | 3493579 | Lu13_5544664 | Lu13_3493579 | 4.425 |  | 2.02 |
| 111 | RTM-GWAS | 91 | FW_MN2019 | 13 |  | 5544664 | 5544664 | Lu13_5544664 |  |  |  | 1.24 |
| 112 | RTM-GWAS | 92 | FW-SK2019 | 13 |  | 19544423 | 19544423 | Lu13_19544423 |  |  |  | 1.54 |
| 113 | RTM-GWAS | 93 | FW_SK2021 | 14 |  | 2166252 | 2166252 | Lu14_2166252 |  |  |  | 1.15 |
| 114 | ICIM-ADD | 25 | FW_MN2019 | 14 | 45 | 3181022 | 3311895 | Lu14_3181022 | Lu14_3311895 | 3.5497 |  | 1.03 |
| 114 | ICIM-ADD | 25 | FW-SK2020 | 14 | 46 | 3181022 | 3311895 | Lu14_3181022 | Lu14_3311895 | 5.7071 |  | 2.30 |
| 114 | ICIM-ADD | 25 | FW_SK2021 | 14 | 46 | 3181022 | 3311895 | Lu14_3181022 | Lu14_3311895 | 10.7407 |  | 5.39 |
| 114 | ICIM-ADD | 25 | FW_mean | 14 | 46 | 3181022 | 3311895 | Lu14_3181022 | Lu14_3311895 | 11.3744 |  | 2.60 |
| 114 | ICIM-ADD | 25 | FW_BLUP | 14 | 46 | 3181022 | 3311895 | Lu14_3181022 | Lu14_3311895 | 10.592 |  | 2.85 |
| 114 | RTM-GWAS | 94 | FW-SK2019 | 14 |  | 3181022 | 3181022 | Lu14_3181022 |  |  |  | 1.55 |
| 115 | ICIM-ADD | 26 | FW-SK2019 | 14 | 48 | 3311895 | 3623917 | Lu14_3311895 | Lu14_3623917 | 10.114 |  | 4.49 |
| 115 | 3VmrMLM | 16 | FW-SK2020 | 14 |  | 3311895 |  | Lu14_3311895 |  |  | 1.95E-09 | 3.19 |
| 115 | 3VmrMLM | 16 | FW-SK2019 | 14 |  | 3311895 |  | Lu14_3311895 |  |  | 0 | 4.13 |
| 115 | 3VmrMLM | 16 | FW_SK2021 | 14 |  | 3311895 |  | Lu14_3311895 |  |  | 0 | 2.88 |
| 115 | 3VmrMLM | 16 | FW_mean | 14 |  | 3311895 |  | Lu14_3311895 |  |  | 1.95E-10 | 2.11 |
| 115 | 3VmrMLM | 16 | FW_BLUP | 14 |  | 3311895 |  | Lu14_3311895 |  |  | 0 | 2.29 |
| 115 | RTM-GWAS | 95 | FW-SK2020 | 14 |  | 3311895 | 3311895 | Lu14_3311895 |  |  |  | 4.18 |
| 115 | RTM-GWAS | 95 | FW-SK2019 | 14 |  | 3311895 | 3311895 | Lu14_3311895 |  |  |  | 5.91 |
| 115 | RTM-GWAS | 95 | FW_MN2019 | 14 |  | 3311895 | 3311895 | Lu14_3311895 |  |  |  | 0.46 |
| 115 | RTM-GWAS | 95 | FW_SK2021 | 14 |  | 3311895 | 3311895 | Lu14_3311895 |  |  |  | 3.19 |
| 116 | RTM-GWAS | 96 | FW_mean | 14 |  | 4035341 | 4035341 | Lu14_4035341 |  |  |  | 2.04 |
| 116 | RTM-GWAS | 96 | FW_BLUP | 14 |  | 4035341 | 4035341 | Lu14_4035341 |  |  |  | 1.92 |
| 117 | RTM-GWAS | 97 | FW_BLUP | 14 |  | 4417462 | 4592734 | Lu14_LDB_4417462_4592734 |  |  |  | 0.82 |
| 118 | RTM-GWAS | 98 | FW_SK2021 | 14 |  | 14725855 | 14789075 | Lu14_LDB_14725855_14789075 |  |  |  | 1.35 |
| 119 | RTM-GWAS | 99 | FW-SK2020 | 14 |  | 14987432 | 14987432 | Lu14_14987432 |  |  |  | 1.72 |
| 120 | 3VmrMLM | 17 | FW_MN2019 | 14 |  | 15760206 |  | Lu14_15760206 |  |  | 1.37E-08 | 2.00 |
| 120 | RTM-GWAS | 100 | FW_MN2019 | 14 |  | 15760206 | 15760206 | Lu14_15760206 |  |  |  | 1.67 |
| 120 | RTM-GWAS | 100 | FW_mean | 14 |  | 15760206 | 15760206 | Lu14_15760206 |  |  |  | 0.95 |
| 121 | RTM-GWAS | 101 | FW_BLUP | 14 |  | 16575028 | 16575028 | Lu14_16575028 |  |  |  | 0.56 |
| 122 | RTM-GWAS | 102 | FW_SK2021 | 14 |  | 18684431 | 18684431 | Lu14_18684431 |  |  |  | 0.56 |
| 123 | RTM-GWAS | 103 | FW_MN2019 | 15 |  | 9950478 | 9950685 | Lu15_LDB_9950478_9950685 |  |  |  | 0.67 |
| 123 | RTM-GWAS | 103 | FW_mean | 15 |  | 9950478 | 9950685 | Lu15_LDB_9950478_9950685 |  |  |  | 0.38 |
| 124 | RTM-GWAS | 104 | FW-MN2021 | 15 |  | 10500592 | 10500592 | Lu15_10500592 |  |  |  | 2.06 |
| 125 | 3VmrMLM | 18 | FW-SK2020 | 15 |  | 12157884 |  | Lu15_12157884 |  |  | 5.02E-07 | 2.22 |
| 126 | RTM-GWAS | 105 | FW-SK2020 | 15 |  | 12509434 | 12509518 | Lu15_LDB_12509434_12509518 |  |  |  | 2.75 |
| 127 | RTM-GWAS | 106 | FW_SK2021 | 15 |  | 13906725 | 14056095 | Lu15_LDB_13906725_14056095 |  |  |  | 1.04 |
| 128 | RTM-GWAS | 107 | FW_mean | 15 |  | 14297856 | 14297856 | Lu15_14297856 |  |  |  | 0.67 |
| 129 | RTM-GWAS | 108 | FW_SK2021 | 15 |  | 14448209 | 14448251 | Lu15_LDB_14448209_14448251 |  |  |  | 1.11 |
| 129 | RTM-GWAS | 108 | FW_BLUP | 15 |  | 14448209 | 14448251 | Lu15_LDB_14448209_14448251 |  |  |  | 0.70 |

^a^ 129 QTLs were identified with all three models combined; ^b^ 24 QTLs were identified with the ICIM-ADD model implemented in IciMapping, 18 QTNs with 3VmrMLM implemented in IIIVmrMLM and 108 LD blocks with RTM-GWAS; ^c^ LOD: logarithm of odds score associated with significant QTLs detected by ICIM-ADD; ^d^ PVE: percent of the variance explained

Table S3 List of candidate genes for Fusarium wilt resistance identified in the quantitative trait locus (QTL) interval Lu1_1,769,377-2,636,369 on chromosome 1 of ‘CDC Bethune’ reference assembly v2.0 (You et al. 2018). Gene ID, predicted protein annotation, gene position and orientation (+ or – strand) in the ‘Bison’ assembly (tigBison) generated using PacBio high-fidelity (HiFi) circular consensus sequence (CCS) reads.

| **Gene ID** | **Annotation** | **Orientation and position in tigBison^a^ chromosome 1** |
| --- | --- | --- |
| *Lus10025849* | LRR receptor-like serine-threonine protein kinase | (-) 2,051,606-2,055,405 |
| *Lus10025852* | Protein suppressor of npr-1 or TMV resistant protein N-like | (-) 2,061,474-2,063,279 |
| *Lus10025882* | Serine-threonine protein kinase OXI1 | (-) 2,189,696-2,191,122 |
| *Lus10025891* | G-type lectin or receptor-like serine-threonine kinase | (+) 2,222,846-2,225,723 |
| *Lus10025926* | LRR receptor-like serine-threonine protein kinase | (-) 2,351,825-2,356,719 |
| *Lus10025941* | Serine-threonine protein kinase | (-) 2,401,851-2,406,330 |

^a^ tigBison Pac-Bio HiFi assembly

Table S4 List of single nucleotide polymorphism (SNP) and Kompetitive allele-specific PCR (KASP) markers located at the major Fusarium wilt quantitative trait locus (QTL) of chromosome 1 of flax. Marker ID, maker type, allele (Nuc), SNP position in GenBank accession CP127619 (chromosome 1 of ‘CDC Bethune’ reference assembly v2.0) and in the ‘Bison’ assembly, gene ID and annotation are listed.

| **Marker ID** | **Type^a^** | **Nuc^b^** | **Position in CP027619** | **Position in tigBison** | **Gene ID** | **Annotation** |
| --- | --- | --- | --- | --- | --- | --- |
| Lu1_1769377 | SNP | G/A | 1769377 | 1976098 |  | Intergenic |
| Lu1_1779713 | KASP | C/T | 1779713 | 1986337 |  | Intergenic |
| Lu1_1782718 | KASP | A/G | 1782718 | 1989496 | *Lus10025835* | Hypothethical uncharacterized protein |
| Lu1_1783307 | SNP | A/G | 1783307 | 1990085 | *Lus10025835* | Hypothethical uncharacterized protein |
| Lu1_1816491 | KASP | A/G | 1816491 | 2022720 | *Lus10025843* | DNA repair protein RadA-like |
| Lu1_1915731 | SNP | T/G | 1915731 | 2121310 | *Lus10025861* | DUF538 domain-containing protein |
| Lu1_1924650 | KASP | G/T | 1924650 | 2130230 | *Lus10025865* | Fimbrin-2 |
| Lu1_1951670 | KASP | A/G | 1951670 | 2152842 | *Lus10025870* | Abasic site processing protein |
| Lu1_1951798 | SNP | T/C | 1951798 | 2152970 | *Lus10025870* | Abasic site processing protein |
| Lu1_1951926 | SNP | T/C | 1951926 | 2153098 | *Lus10025870* | Abasic site processing protein |
| Lu1_1951991 | SNP | G/A | 1951991 | 2153163 | *Lus10025870* | Abasic site processing protein |
| Lu1_1966369 | SNP/KASP | T/A | 1966369 | 2168010 | *Lus10025875* | Cystein-rich repeat secretory protein 55-like |
| Lu1_1982778 | KASP | C/T | 1982778 | 2184432 | *Lus10025881* | Protein NRTI/PTR family 4.2 like |
| Lu1_2021549_ind | KASP | ------/ TTGCTG | 2021549 | 2223295 | *Lus10025891* | G-type lectin S-receptor-like serine/threonine-protein kinase |
| Lu1_2021554_G | KASP | G/A | 2021554 | 2223300 | *Lus10025891* | G-type lectin S-receptor-like serine/threonine-protein kinase |
| Lu1_2448006 | KASP | A/T | 2448006 | 2249670 | *Lus10025900* | Hypothethical uncharacterized protein |
| Lu1_2470015 | KASP | A/C | 2470015 | 2271711 | *Lus10025905* | DNA ligase 1 |
| Lu1_2473043 | KASP | T/C | 2473043 | 2274728 | *Lus10025906* | Hypothethical uncharacterized protein |
| Lu1_2493432 | KASP | A/T | 2493432 | 2295483 | *Lus10025909* | Indole-3-acetic acid-induced protein ARG7 |
| Lu1_2500556 | KASP | G/T | 2500556 | 2302607 | *Lus10025913* | B3 domain-containing transcription factor VRN1 |
| Lu1_2500703 | SNP | T/C | 2500703 | 2302756 | *Lus10025913* | B3 domain-containing transcription factor VRN1 |
| Lu1_2530312 | KASP | G/C | 2530312 | 2332395 | *Lus10025922* | Myrcene synthase, chloroplastic-like |
| Lu1_2531543 | SNP | A/G | 2531543 | 2333605 | *Lus10025922* | Myrcene synthase, chloroplastic-like |
| Lu1_2540586 | SNP | A/C | 2540586 | 2342648 |  | Intergenic |
| Lu1_2544098 | SNP | C/A | 2544098 | 2346173 | *Lus10027979* | CALCOFLUOR WHITE HYPERSENSITIVE PROTEIN PRECURSOR |
| Lu1_2636369 | SNP | A/C | 2636369 | 2420368 |  | Intergenic |

^a^ SNPs were identified through read mapping and filtering of the genotyping-by-sequencing reads of the ‘Bison’/’Novelty’ recombinant inbred lines; ^b^ Nuc: nucleotide
